# An Amino‐Yne Click Chemistry Approach for Multi‐Responsive Liquid Crystal Elastomer Actuators

**DOI:** 10.1002/smll.202509070

**Published:** 2025-10-28

**Authors:** Sara Bescós‐Ramo, Marco Turriani, Camilla Parmeggiani, Milagros Piñol, Luis Oriol, Daniele Martella

**Affiliations:** ^1^ Instituto de Nanociencia y Materiales de Aragón (INMA) CSIC‐Universidad de Zaragoza Zaragoza 50009 Spain; ^2^ Departamento de Química Orgánica Facultad de Ciencias Universidad de Zaragoza Zaragoza 50009 Spain; ^3^ European Laboratory for Non‐Linear Spectroscopy (LENS) Via N. Carrara 1 Sesto Fiorentino 50019 Italy; ^4^ Department of Physics and Astronomy University of Florence Via S. Sansone 1 Sesto Fiorentino 50019 Italy; ^5^ Department of Chemistry “Ugo Schiff” University of Florence Via della Lastruccia 3–13 Sesto Fiorentino 50019 Italy

**Keywords:** amino‐yne, click chemistry, liquid crystal elastomers, soft actuator, stimuli‐responsive polymers

## Abstract

The spontaneous amino‐yne click reaction is introduced for the first time as a fast, efficient, and ambient temperature strategy for preparing multi‐responsive Liquid Crystal Elastomer (LCE) actuators. This synthetic approach relies on the amino‐yne cross‐linking between secondary amines along the main chain of poly(β‐amino ester) liquid crystal oligomers and a dipropiolate‐functionalized cross‐linker, rendering β‐aminoacrylate cross‐linking points. After alignment and locking via dynamic transesterification at 30 °C, the resulting LCE actuators exhibit reversible and reproducible thermal and light‐induced actuation. The generated tension and activation kinetics are easily tunable by adjusting the cross‐linker content. Additionally, leveraging the acid‐triggered reactivity of the network enables it to respond to additional stimuli, including water‐driven actuation. By integrating the capability for dynamic bond exchange, this LCE formulation opens new avenues for designing structures with complex molecular orientation patterns. As a proof of concept, a star‐shaped soft actuator is fabricated, demonstrating diverse programmable actuation modes.

## Graphical abstract

The spontaneous amino‐yne click reaction is introduced as a fast, efficient, and ambient temperature strategy for preparing multi‐responsive Liquid Crystal Elastomer (LCE) actuators. The materials show reversible thermal and light‐induced actuation, as well as water‐driven actuation thanks to network's acid‐triggered reactivity. By integrating dynamic bond exchange, this LCE formulation opens new pathways for complex orientation patterning.

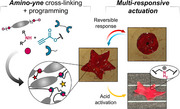

## 1 Introduction

Amino‐yne click chemistry refers to the spontaneous reaction between amines and electron‐deficient alkynes, such as propiolates, proceeding under ambient conditions and within short reaction times, without the need for catalysts.^[^ ^1^ ^]^ This transformation quantitatively yields β‐aminoacrylate bonds, which are cleavable in acidic media.^[^ ^2^ ^]^ The ease of implementation, mild reaction conditions, and high reaction yields, fulfill many of the criteria defined for click reactions. Consequently, this chemistry has become a promising approach for preparing functional materials since the first report of spontaneous amino‐yne click polymerization in 2017.^[^ ^3^ ^]^ More recently, it has found applications across various areas of polymer science, including sequence‐controlled polymers,^[^ ^4^ ^]^ vitrimers,^[^ ^5^ ^]^ thermosets,^[^ ^6^ ^]^ hydrogels,^[^ ^7^, ^8^, ^9^ ^]^ drug delivery systems,^[^ ^10^ ^]^ and more. Nevertheless, its potential for application in the field of polymeric actuators, materials capable of performing mechanical work in response to external stimuli, remains unexplored.

Liquid crystal elastomers (LCEs) are cross‐linked polymer networks that combine the large deformability of elastomeric systems with the anisotropic self‐organization of liquid crystals.^[^ ^11^, ^12^ ^]^ Stimuli‐responsive LCEs have emerged as promising candidates for soft actuators in applications such as robotics,^[^ ^13^, ^14^ ^]^ sensing,^[^ ^15^, ^16^ ^]^ and tissue engineering.^[^ ^17^, ^18^ ^]^ In their equilibrium configuration, LCEs typically exhibit a polydomain state, which lacks macroscopic order. However, precise actuation requires a uniform alignment of mesogens throughout the material. External stimuli (traditionally temperature, but also light,^[^ ^16^, ^19^, ^20^ ^]^ electric fields,^[^ ^21^ ^]^ or chemical agents^[^ ^22^ ^]^) can induce the liquid crystal to isotropic phase transition, leading to controlled and reversible shape changes. Most recent work in the field has focused on nematically aligned LCEs, which typically exhibit contraction along the nematic director and expansion in the perpendicular direction, in contrast to other types of LCE‐based actuators. Consequently, synthesizing LCEs with controlled liquid crystal alignment is essential for unlocking their capabilities.^[^ ^23^ ^]^

Although cell alignment techniques are well established, they are primarily applicable to thin films. 3D printing offers an attractive alternative, but remains limited by ink viscosity and the challenging mesogen alignment along the printing flow. Mechanical alignment, in contrast, provides a more versatile strategy across different compositions and thicknesses.^[^ ^24^ ^]^ It essentially involves a two‐step process: first, partially cross‐linked liquid crystalline polymers are subjected to strain to orient the mesogenic units along the stretching direction; second, a subsequent reaction completes the cross‐linking of the material, thereby locking the alignment. However, the fragility of partially cross‐linked samples, along with the challenges of inhomogeneous cross‐linking during the second stage (often via UV) limit scalability. Alternatively, mechanical alignment of fully cross‐linked polydomain samples has been achieved through the incorporation of dynamic covalent bonds, which enable bond exchange upon exposure to external stimuli.^[^ ^25^, ^26^ ^]^ Upon removal of the stimulus, the material retains the mechanically programmed orientation, allowing the fabrication of actuators from fully cross‐linked networks.

From the perspective of the initial cross‐linking stage, traditional chemistries for LCE synthesis, such as hydrosilylation, free radical polymerization of reactive mesogens or polyaddition between epoxy and carboxylic acid‐functional monomers, often require long reaction times, lack control over the network structure, or necessitate high temperatures to proceed.^[^ ^24^, ^27^, ^28^ ^]^ To address these challenges, recent attention has been focused on click chemistries, which enable a more precise control over the structures and properties of LCEs.^[^ ^29^ ^]^ A wide range of click‐like reactions have been applied to date for the fabrication of LCE actuators. In particular, base‐catalyzed thia‐Michael and aza‐Michael additions to acrylates have been extensively exploited,^[^ ^30^, ^31^, ^32^, ^33^ ^]^ along with radical‐mediated thiol‐ene and thiol‐yne reactions,^[^ ^34^, ^35^, ^36^ ^]^ base‐catalyzed thiol‐epoxy reactions,^[^ ^37^ ^]^ Diels‐Alder cycloaddition,^[^ ^38^, ^39^ ^]^ and copper‐catalyzed azide‐alkyne cycloaddition (CuAAC).^[^ ^40^ ^]^ Nevertheless, the aforementioned amino‐yne chemistry has not yet been explored for the preparation of LCEs, despite offering several advantages.

The use of conjugate addition of nucleophiles such as amines for LCE fabrication was pioneered by Ware et al.,^[^ ^32^ ^]^ who developed an approach that combines aza‐Michael addition with free‐radical polymerization of acrylate‐terminated liquid crystal oligomers (LCO). Years later, Zou et al.^[^ ^33^ ^]^ demonstrated a method for fabricating monodomain LCEs based solely on amine‐acrylate aza‐Michael addition, without the need for UV photocross‐linking. Their approach involves a two‐step synthesis that exploits the significant kinetic difference between the fast addition of primary amines and the slower reaction of the resulting secondary amines with acrylates. However, in contrast to their system, which requires elevated temperatures (90 °C) to promote secondary amine reactivity, the amino‐yne reaction can proceed spontaneously at room temperature and without any catalyst. This milder and more straightforward process reduces the likelihood of side reactions and offers greater control over network formation. These features make amino‐yne chemistry not only a promising strategy for network cross‐linking, but also a more environmentally friendly alternative to more classical click reactions such as CuAAC, which typically require metal catalysts and elevated temperatures.

**Figure 1:**
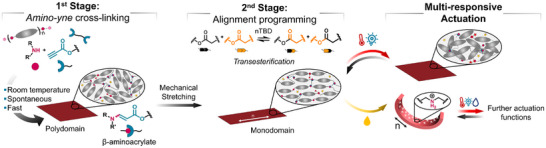
Schematic representation of the two‐stage preparation of β‐aminoacrylate‐based LCE films and their programmable multi‐responsive actuation.

Herein, the key objective of this work is to explore the amino‐yne spontaneous cross‐linking approach for the efficient, ambient temperature preparation of multi‐responsive β‐aminoacrylate‐based LCE actuators. Accordingly, we first synthesized polydomain LCEs via amino‐yne cross‐linking of secondary amine‐based LCOs and a dipropiolate cross‐linker (**Figure** **1**, 1st stage). After systematically investigating the influence of the cross‐linker and mesogenic content on the thermal and thermomechanical properties, the second step involved mechanical aligning of the mesogenic units and fixing this alignment through dynamic covalent bond exchange reactions (Figure 1, 2nd stage). Following the mild‐condition principles of click chemistry, this exchange step can be carried out using a recently reported base‐catalyzed transesterification method at near‐ambient temperature (30 °C),^[^ ^41^ ^]^ which enables the fabrication of mechanical actuators with complex orientation patterns that exhibit reversible responses to stimuli such as temperature and light (Figure 1, Multi‐responsive Actuation). By further exploiting the acid‐triggered reactivity of amino‐based structures, we can obtain alternative pathways for material programming and additional stimuli‐responsive behaviors. As a proof of concept for the versatility of this approach, a water‐triggered response was demonstrated.

## 2 Results and Discussion

### 2.1 Design, Preparation, and Characterization of β‐Aminoacrylate‐Based LCEs

**Figure 2:**
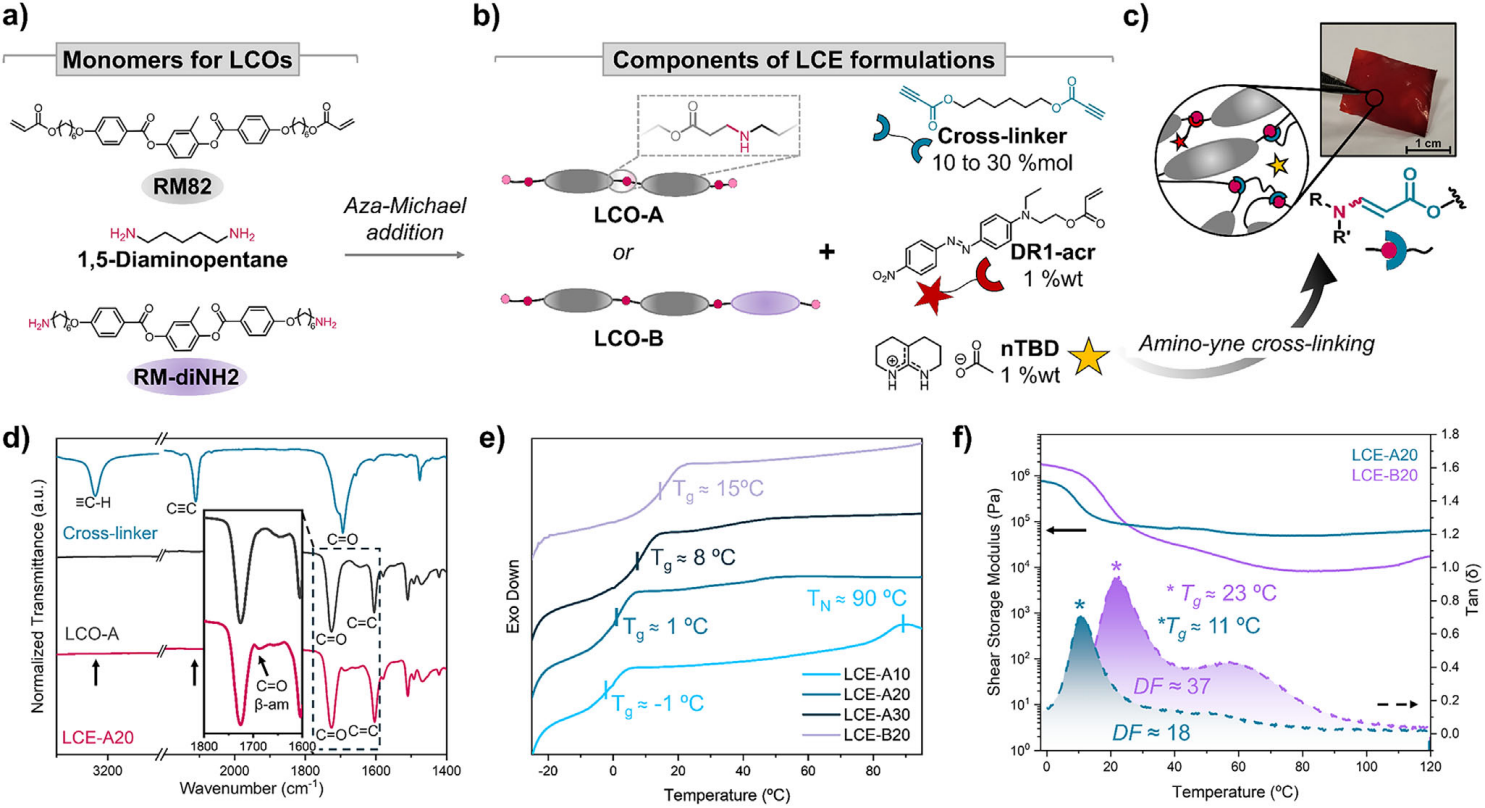
Synthesis and characterization of amino‐yne cross‐linked LCEs. 1st Stage of material preparation, network formation. a) Synthetic scheme showing monomers for LCOs and b) components of LCE formulations. c) Image showing self‐standing polydomain **LCE‐A20** film after amino‐yne cross‐linking, d) ATR spectra of **LCE‐A20** (below), **LCO‐A** (middle), and dipropiolate cross‐linker (above). e) DSC curves of **LCE‐A** with different mol% of cross‐linker (from 10 to 30) and **LCE‐B20**. *T_g,DSC_* was determined from the midpoint of the baseline jump during the second heating cycle of the sample with a scan rate = 20 °C min^−1^. f) **LCE‐A20** and **LCE‐B20** DMA curves of shear storage modulus (solid lines) and *tan δ* (dashed lines) as a function of temperature. *T_g,DMA_* was determined as the temperature corresponding to the maximum of *tan δ* curves. The dissipation factor (DF) was determined as the area under the *tan δ* curve between *T_g_* – 30 °C (for **LCE‐A20**) or *T_g_* – 20 °C (for **LCE‐B20**) and 120 °C (scan rate = 2 °C min^−1^, frequency = 1 Hz, and parallel plate geometry).

Our strategy relies on the spontaneous amino‐yne cross‐linking between secondary amines located along the main chain of poly(β‐amino ester) LCOs and a dipropiolate‐functionalized cross‐linker (Scheme S1, Figures S1 and S2, Supporting Information). Accordingly, two LCOs were first synthesized via aza‐Michael step‐growth polymerization at room temperature using an excess of amine groups, ensuring that oligomer formation occurs mainly through reactions involving primary amines (see monomer structures in **Figure** **2**a). For the synthesis of **LCO‐A**, a commercially available diacrylate LC nematic monomer (**RM82**) and a monomeric diamine (1,5‐diaminopentane) were used in a 1:1.1 molar ratio (diamine excess). **LCO‐B** was prepared by combining **RM82** with 1,5‐diaminopentane and a synthesized LC nematic diamino monomer referred to as **RM‐diNH2** (Figure 2a) in a 1:0:55:0:55 molar ratio (50% of each diamino monomer). Synthesis and characterization of **RM‐diNH2** are reported in the Supporting Information (Scheme S2, Figures S3–S8, Supporting Information). Synthetic details for LCO preparation are reported in Supporting Information. ^1^H NMR analysis confirmed the complete conversion of the acrylate groups for both LCOs (Figures S9 and S10, Supporting Information). Therefore, the theoretical degree of polymerization was calculated as approximately 10–11, according to Carothers’ equation (Equations S1–S3 and Table S1, Supporting Information).^[^ ^42^ ^]^ Using ^1^H NMR end group analysis, experimental degree of polymerization of **LCO‐A** was determined to be ≈10 (Figure S9 and Equation S4, Supporting Information); however, the lack of well‐defined end‐group proton signals in **LCO‐B** prevented its experimental estimation. Traces of minor reactions, attributed to secondary amine‐initiated conjugate addition or amidation, were observed in the spectra, consistent with previous reports for aza‐Michael step‐growth polymerizations (Figure S11, Supporting Information).^[^ ^43^ ^]^ Their mesomorphic properties were investigated by Differential Scanning Calorimetry (DSC) and Polarized Optical Microscopy (POM) (Figures S12 and S13, Supporting Information), observing that oligomers exhibited liquid crystalline texture typical of the nematic mesophase. The nematic‐to‐isotropic liquid transition temperature (*T_NI_*) was found to be higher for **LCO‐B** (124 °C) than for **LCO‐A** (82 °C), which is in accordance with the higher rigidity of the former.

As previously discussed, the preparation of β‐aminoacrylate‐based LCE films involves two‐stages, network formation by amino‐yne cross‐linking and locking the mechanical alignment by dynamic covalent chemistry (Figure 1). For the first stage, two dichloromethane solutions were prepared with the components of the LCE formulation (Figure 2b): one containing the oligomer **LCO‐A** or **LCO‐B**, and the other containing the dipropiolate cross‐linker, Disperse Red 1 acrylate (**DR1‐acr**), and neutralized triazabicyclodecene (**nTBD**). **DR1‐acr** becomes incorporated into the network via aza‐Michael reaction with the amino groups present in the LCOs and enables light‐induced control over the material's deformation.^[^ ^12^, ^19^ ^]^ Meanwhile, **nTBD** (prepared as reported in Supporting Information) was added as a catalyst for the transesterification bond exchange reaction required in the second stage of material preparation (Figure 1, 2nd Stage).^[^ ^41^, ^44^ ^]^ The solutions were combined and homogeneously mixed immediately before being cast onto a PVA‐covered glass substrate at room temperature. A polymeric film was formed within a few seconds after casting and was left to dry at room temperature overnight prior to characterization. A series of three LCEs were prepared using **LCO‐A** and different dipropiolate molar percentage (10, 20, and 30 mol%), to investigate how the LCE properties are affected by the amount of cross‐linker (Tables S2 and S3, Supporting Information). Instead, only 20 mol% of cross‐linker was used for preparing an LCE with **LCO‐B**. Throughout this article, β‐aminoacrylate‐based LCEs are denoted as LCE‐*xy*, where *x* represents the LCO used (*x* = A or B) and *y* the mol% of cross‐linker used in the LCE.

Spontaneous amino‐yne reaction between secondary amines in LCOs’ main chain and propiolate groups enabled the formation of cross‐linked networks, yielding polydomain LCE films (Figure 2c). To confirm that the samples were fully cross‐linked, gel content tests were performed to quantify the fraction of the material forming an insoluble network (see Characterization Techniques in Supporting Information). In all cases, the gel content exceeded 94%, indicating the efficiency of the cross‐linking strategy. Taking **LCE‐A20** as an example, Attenuated Total Reflection (ATR) infrared spectroscopy showed that typical signals assigned to the propiolate^[^ ^3^ ^]^ (Figure 2d, ≡C−H and C≡C stretching vibrations at 3225 and 2110 cm^−1^, respectively) disappeared in the cross‐linked sample, indicating high conversion ratio. Moreover, a band associated with C = O stretching vibrations of β‐aminoacrylate appeared at 1689 cm^−1^ if compared to the **LCO‐A** spectrum, revealing the formation of the cross‐linking group.

The glass transition temperatures measured by DSC analysis (*T_g,DSC_*) for the different films are reported in Figure 2e. As expected, the cross‐linking increase led to a corresponding increase in *T_g_*, from ‐1 °C in **LCE‐A10** to 8 °C in **LCE‐A30**. A second transition, assigned to the *T_NI_*, was only perceptible around 90 °C for the sample with the lowest cross‐linker content, which is consistent with previous literature.^[^ ^45^ ^]^ Moreover, the incorporation of the **RM‐diNH2** in **LCE‐B20**, and consequent increase of rigidity due to the higher content of mesogenic monomers in the mixture, was accompanied with a notorious increase of *T_g_* with respect to **LCE‐A20** (15 and 1 °C, respectively). Thermal properties of LC monomers and LCOs are collected in Table S4 (Supporting Information).

The thermomechanical properties of the LCE networks were investigated by Dynamic Mechanical Analysis (DMA) in the shear mode on polydomain samples. Results compiled in Figure 2f and Figure S14 (Supporting Information) illustrate different thermal regimes with their corresponding mechanical properties. Below glass transition (*T_g,DMA_*), materials show a glassy state with comparable *G’* storage in all cases (0.4‐0.7 MPa for **LCE‐A10/20/30**, and 1.7 MPa for **LCE‐B20**); nevertheless, no clear correlation between *G’* and the amount of cross‐linker was observed in this region (Figure S15, Supporting Information). As temperature increases, the decline in *G’* to the rubbery state leads to a reduction in the moduli, reaching values one order of magnitude lower at 115 °C for **LCE‐A10/20/30** (0.06‐0.03 MPa) and two order for **LCE‐B20** (0.02 MPa). The incorporation of **RM‐diNH2** into the LCE mixture therefore has a greater influence on the modulus than the amount of dipropiolate cross‐linker used. *T_g,DMA_* mimics the tendency observed in DSC results, increasing with the content of cross‐linker and material stiffness (Figure 2f; Figure S14, Supporting Information). Furthermore, as expected for LCEs, a second peak in *tan δ* and a decrease in *G’* indicated the nematic to isotropic transition in the rubbery region. The great width of this transition prevents establishing a clear *T_NI_*, defined as the lowest point of *G*’. However, for all the samples, *tan δ* reverted to ≈0 upon heating above 70–90 °C, suggesting the loss of nematic order (Figure S14, Supporting Information).^[^ ^46^, ^47^ ^]^ The magnitude of *tan δ* also indicates the energy absorption capacity of the films across different temperature ranges. As is commonly observed in this type of materials, elevated *tan δ* in between *T_g_* and *T_NI_* suggests an improvement of energy dissipation in this range. This behavior can be attributed to the increased mobility of the polymer network within the nematic phase, where rotation of mesogenic segments and the viscous deformation of polymeric chains contribute to energy dissipation. The ability to absorb energy of the films can therefore be quantified by the dissipation factor, calculated as the area under *tan δ*.^[^ ^48^ ^]^ In this case, it increases slightly with mol% of cross‐linker (Figure S14, Supporting Information) but more sharply when comparing **LCE‐A20** and **LCE‐B20** (Figure 2f). Notably, **LCE‐B20** exhibits a high loss factor (exceeding 0.9) in the 20–25 °C range, which indicates significant damping properties at ambient temperatures.

Further mechanical characterization have been performed on selected samples by tensile test, reported in Figure S16 and Table S5 (Supporting Information). Indeed, **LCE‐A30** demonstrates both high stress at break and strain at break (9.1 ± 0.4 MPa and 126 ± 19% respectively) and, as expected, a lower cross‐linker content decreases the first parameter while increasing the former one.

### 2.2 Programmability of Soft Actuators and Thermal Actuation

**Figure 3:**
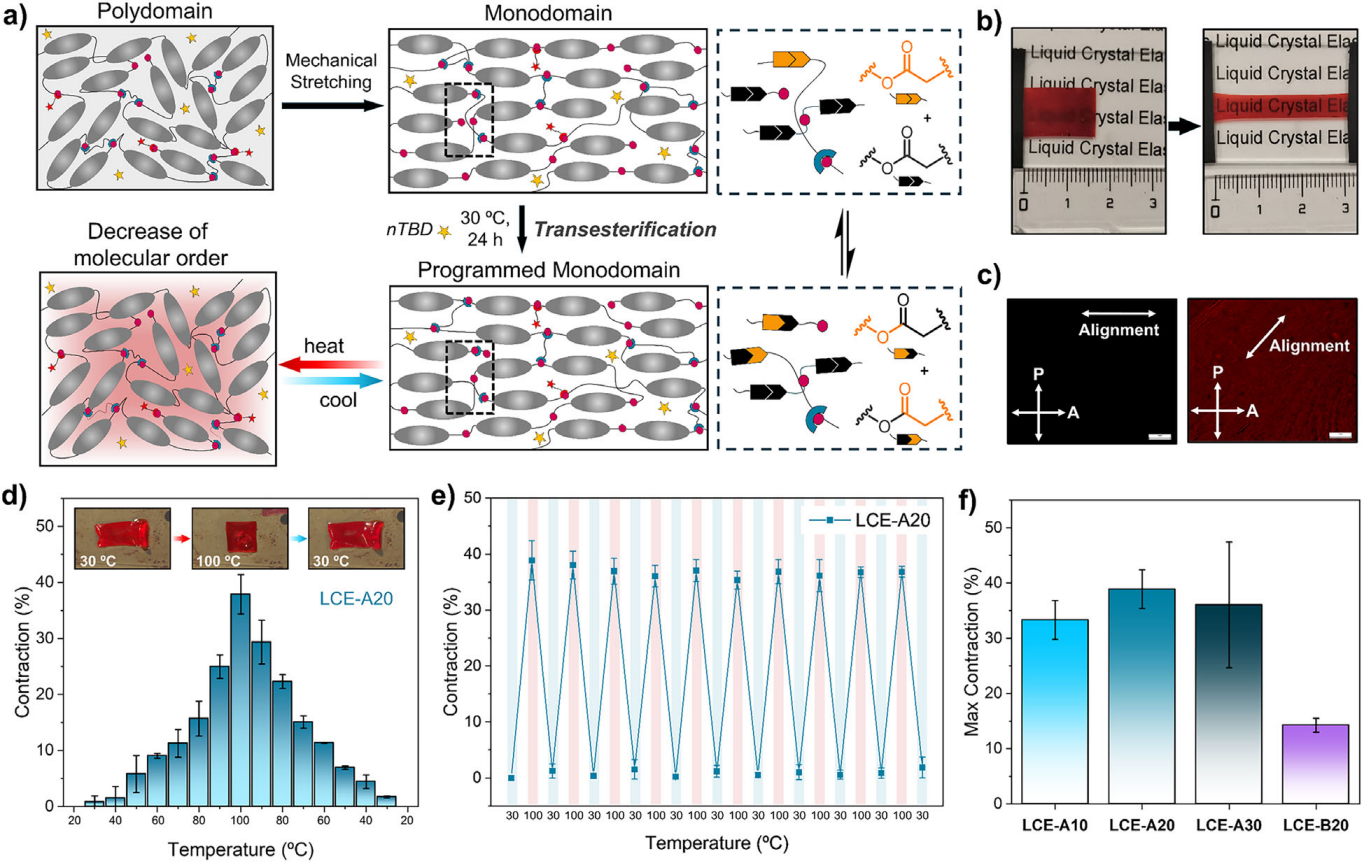
LCE programming and thermal shape‐change. 2nd Stage of material preparation, alignment programming. a) Schematic illustration showing the alignment by mechanical stretching and fixing process by transesterification, and thermal actuation in LCEs, b) images showing polydomain (left) and optically transparent stretched monodomain (right) LCE film, and c) POM images of aligned LCE with the director at 0° (dark) and 45° (bright) to the polarizer. d) Reversible contraction (%) during the first thermal actuation test (from 30 to 100 °C and back) for **LCE‐A20**, e) 10 actuation cycles of **LCE‐A20** between 30 and 100 °C and f) comparison of the maximum contraction (%) recorded for each material at 100 °C for the first actuation cycle. All data is presented as mean of three different films ± SD (n = 3).

The second stage of the material preparation consists of the alignment of the mesogenic units through mechanical stretching and dynamic bond exchange (**Figure** **3**a). In the simplest case, a polydomain film was stretched uniaxially up to 100% of its length, leading to an optically transparent monodomain film (Figure 3b). Then, the sample was kept at near‐ambient temperature (30 °C) to obtain a **nTBD**‐catalyzed transesterification reaction, which allows for the rearrangement of the network without modification of the cross‐linking density. In this way, locking of alignment was achieved, and a new programmed monodomain film was obtained with a thickness of 88 ± 7 µm. After releasing the stretching force, the film underwent a heating‐cooling cycle to eliminate residual deformation and reach equilibrium (see details in Experimental Section). During annealing, the LCE initially contracted upon heating and elongated upon cooling, stabilizing at ≈70% of its pre‐annealing stretched length. Subsequently, the thermal contraction became reversible and reproducible over multiple cycles. POM was used to confirm that the orientation of the mesogenic units was preserved. Figure 3c indicates a uniformly planar alignment of the LCE structure when rotating the sample with respect to the polarizers. Light extinction was observed when the director of the film was orthogonal to the polarizers, and contrast inversion when they formed a 45° angle.

In order to evaluate thermal actuation properties of the materials, free‐standing experiments were carried out by measuring the length of the LCE films during the heating and cooling of the films. Actuation experiments were performed up to a maximum temperature of 100 °C, since it has been previously reported that the ester groups in the RM82‐based LCEs have a tendency to degrade in the presence of unreacted amines when exposed to elevated temperatures (>90 °C) for extended periods.^[^ ^33^ ^]^ The results, given as the percentage of film contraction in Figure 3d, illustrate how the initial uniaxial alignment of the LCE films resulted in the gradual contraction of the films along the nematic director during a heating ramp. When the film was cooled, the reverse behavior was observed, with its length progressively increasing until it reached its initial dimensions at 30 °C. This process has been shown to occur in all the materials (Figure S17, Supporting Information) and to be fully reversible and reproducible after at least 25 heating‐cooling cycles, with no significant loss of actuation capability, as demonstrated in Figure S18 (Supporting Information) and Figure 3e for the first 10 cycles of **LCE‐A20**. The effect on actuation performance of stretch holding time, i.e., the time of maintaining the LCE sample in a stretched state at 30 °C, was also investigated. However, almost no improvement was observed during the first 72 h, and therefore, 24 h was fixed as stretch holding time for programming the materials (Figure S19, Supporting Information).

When comparing the maximum contraction of all the LCEs, no substantial differences were found by increasing the content of cross‐linker in LCE‐Ay (Figure 3f, from 33 to 38%). Nevertheless, obtaining aligned **LCE‐A30** films proved challenging, as some strips broke during the alignment process, when stretched to 100% of their length. Moreover, it was observed a decrease in the maximum contraction for **LCE‐B20** (15%) if compared to LCE‐Ay films, implying that the increase in rigidity, which results from its higher content of aromatic rings and the subsequent reduction in the material flexibility, would restrict the contraction performance of the LCE. These results highlight that, although our materials exhibit lower moduli than those reported for previously tested aza‐Michael LCE films under the same conditions,^[^ ^49^ ^]^ the deformation amplitude measured during thermal actuation in self‐standing films is comparable to or even exceeds previously reported values (23–27%).

### 2.3 Light Actuation

**Figure 4:**
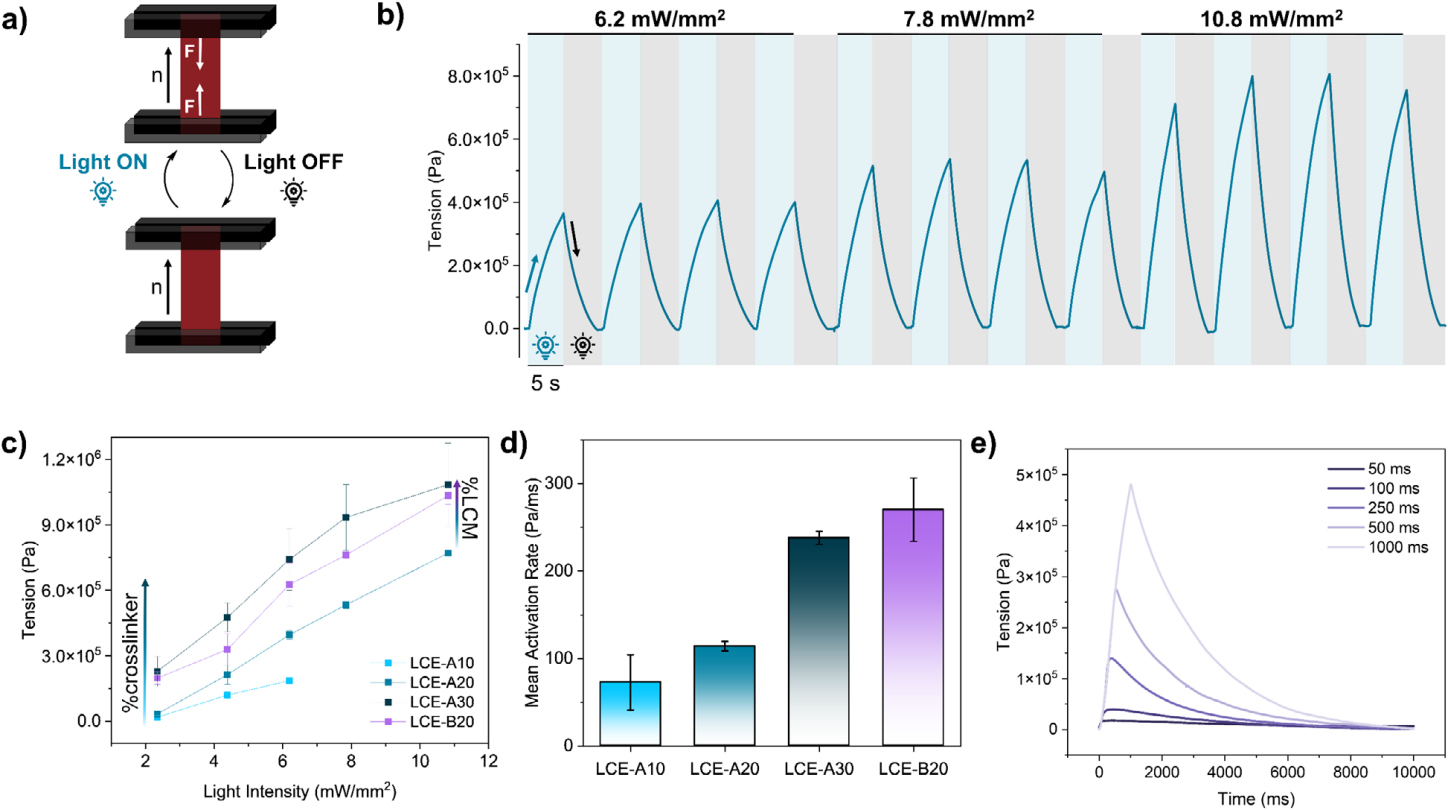
Light Actuation. a) Scheme of light actuation experiment under isometric conditions. The length of the strip is kept constant allowing the measurement of the generated traction force (indicated by white arrows). b) Trace of multiple activation and relaxation cycles of **LCE‐A20** under pulsed irradiation and increasing light intensity (5 s, from 6.2 to 10.6 mW mm^−2^). c) Maximum tension developed by each material under different light intensities after 5 s of irradiation (data is presented as mean ± SD (n = 3)). d) Activation rate at 6.2 mW mm^−2^ (data was obtained as the slope of the tangent at the first 200 ms of the trace of activation, and presented as mean ± SD (n = 3)) and e) tension generated by **LCE‐B20** activated by progressively increasing the illumination time (from 50 to 1000 ms).

The photomechanical response developed by the LCEs was characterized by measuring material's force production under isometric conditions using a customized set‐up (Figure S20, Supporting Information).^[^ ^49^ ^]^ Briefly, LCE films were cut into strips approximately 12 mm in length, 2 mm in width, and 0.09 mm in thickness, with their longest dimension aligned along the nematic director. Then, the strips were mounted vertically under isometric conditions (**Figure** **4**a), with the upper part attached to a force transducer and the LCE strip located between two LED lamps emitting at 𝜆 = 470 nm, overlapping the maximum absorption of **DR1‐acr**.^[^ ^50^ ^]^ The presence of this dye enables material's light‐induced deformation, resulting from both the *trans‐cis* isomerization of the dye and the photothermal effect.^[^ ^12^, ^19^ ^]^

Upon mounting the material, activation and relaxation cycles were recorded under pulsed irradiation. Long pulse experiments were initially performed by illuminating the sample for 5 seconds, followed by 5 seconds of darkness. As illustrated in Figure 4a and demonstrated in Figure 4b, when the light was turned on, the samples began to generate tension along the nematic director, which increased over time. Once the stimulus was removed, the material stopped generating force and fully relaxed within ≈4 seconds. On‐off cycles were fully reproducible, as proved for **LCE‐A20** in Figure 4b. Since light actuation not only depends on the material but also on the illumination conditions, all LCE samples were tested under different light intensities, from 2.4 mW mm^−2^ to the threshold at which each sample failed (Figure 4c). All the samples broke when increasing the light power above 10.8 mW mm^−2^, except for **LCE‐A10**, for which 6.2 mW mm^−2^ was the highest intensity that could be tested. As shown in Figure 4c, tension generated by each LCE increased with light intensity in a nearly linear manner; nevertheless, a plateau region was not attained after 5 seconds, even at the highest intensity. The maximum tension generated by the materials at each light intensity increased with the mol% of cross‐linker so that **LCE‐A10** and **LCE‐A30**, the least and most cross‐linked films, exhibited the lowest and highest tension, respectively (Figure S21, Supporting Information). Furthermore, when comparing **LCE‐A20** with **LCE‐B20**, the latter showed a notably higher tension across the entire range of intensities (0.77 and 1.03 MPa, respectively, taking a light power of 10.8 mW mm^−2^ as an example). This behavior is consistent with previous findings,^[^ ^51^ ^]^ and can be explained by the increased stiffness and rigidity of **LCE‐B20**. These trends were also observed in the activation kinetics, where the activation rate was found to increase with a higher molar percentage of the cross‐linker (Figure 4d). Additionally, **LCE‐B20** exhibited the fastest mean activation rate (270 Pa ms^−1^ at 6.2 mW mm^−^^2^), which is in line with the 0.3 MPa/s previously reported values for LCE films tested under the same custom‐made setup, albeit in that case at a lower light intensity (4.7 mW mm^−^^2^).^[^ ^49^ ^]^ Notably, the maximum tension values of our materials are comparable to those reported at the same light intensity. However, due to the enhanced resistance to breakage of the current strips, it is possible to achieve tension values up to one order of magnitude higher than previous findings by increasing the power of the light source.

Based on all these experiments, **LCE‐A20** proved to be the material with the highest reproducibility across the different measurements, while maintaining good actuation performance, exhibiting twice the maximum tension generated by **LCE‐A10** under the same light intensity. Meanwhile, **LCE‐B20** stands out due to its exceptional performance in terms of the tension generated and actuation rate. Therefore, the latter was selected for studying its behavior under progressively shorter pulse experiments (Figure 4e, from 1000 to 50 ms). As anticipated, peak tension diminished with a reduction in illumination time. Nevertheless, as a proof of concept, **LCE‐B20** was still able to generate a maximum tension of ≈0.14 MPa with an illumination time on the order of 250 ms, a duration and tension comparable to the contraction time observed in human cardiac muscle (>0.1 MPa).^[^ ^52^ ^]^

### 2.4 Additional Actuation Functions and Programmability of Complex Designs for Soft Actuators

**Figure 5:**
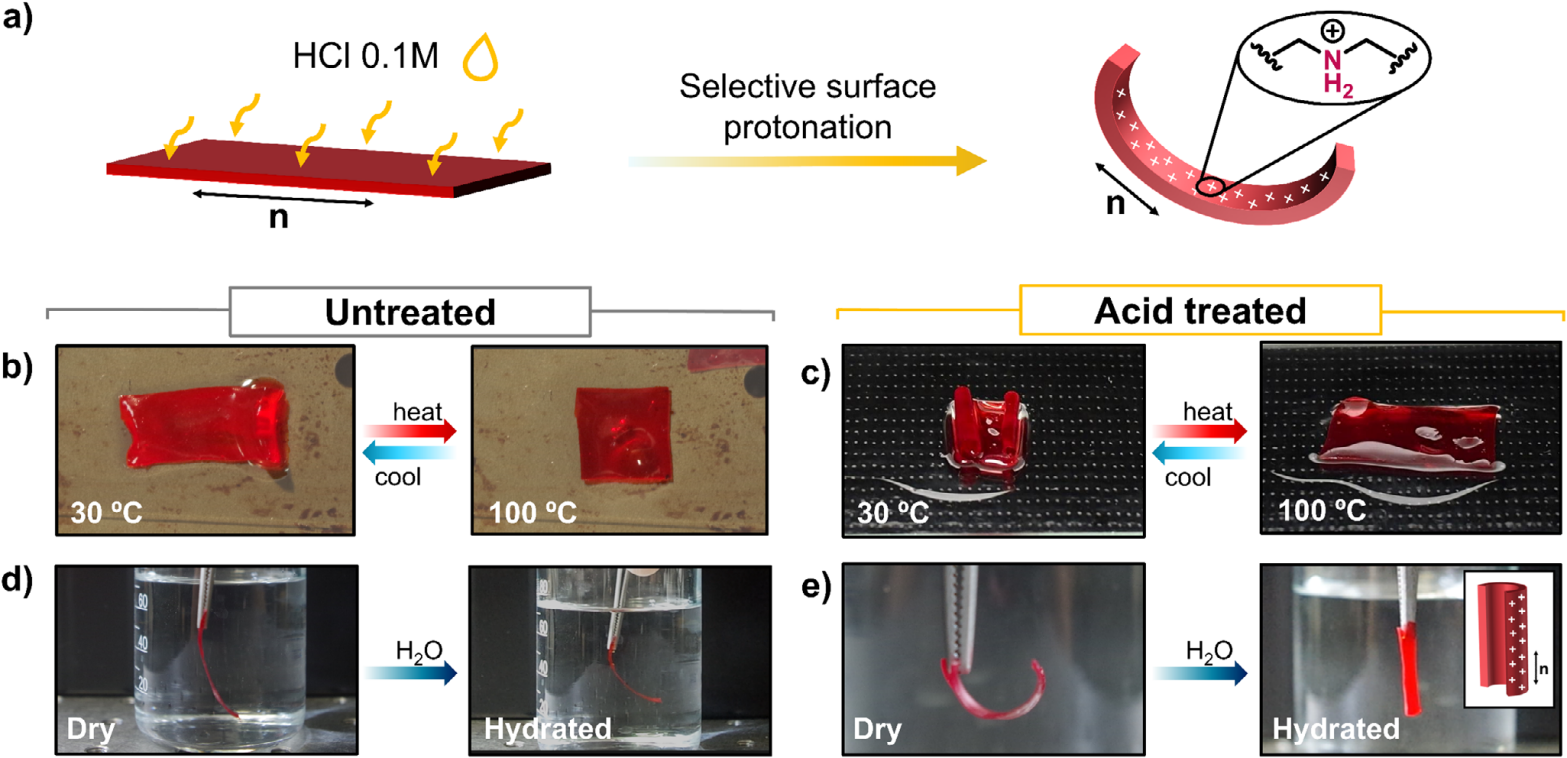
a) Schematic of one‐side **LCE‐A20** film activation by acidic solution. Reversible thermal actuation b) before and c) after acid treatment, demonstrating longitudinal curling upon cooling from 100 to 30 °C of the activated film. The images of the acid‐treated films correspond to the second heating‐cooling cycle, and the activated surface is facing upward. Shape changes before and after immersion in water of d) an untreated film, which shows no actuation upon immersion in water, and e) acid‐treated strip, which forms a tube along the nematic director when hydrated.

Our material, containing both free amino groups and β‐aminoacrylate bonds within its network, opens up further opportunities for actuating the LCE, expanding their shape‐changing capabilities. In particular, the selective acid treatment of one LCE's surfaces provide insights into additional actuations. Among all the samples tested, **LCE‐A20** was selected for studying its behavior after acid treatment, due to its straightforward preparation procedure with only commercial reagents and the efficient and reproducible actuation performance. A rectangular strip was cut to have the long axis parallel to the nematic director. Then, one of its surfaces was activated though exposing it to a 0.1 m hydrochloric acid aqueous solution (see Experimental Section for details). As established in previous studies of amino‐based LCEs, the selective protonation of the amino groups can be achieved (**Figure** **5**a).^[^ ^53^ ^]^ ATR spectrum after acidic activation shows a broad peak at 3500 cm^−1^, which was attributed to moisture on the protonated film surface (Figure S22, Supporting Information).

New deformation behaviors under external stimuli were found for the activated films. Right after acid treatment, the film exhibited a curvature directed toward the treated surface and aligned parallel to the nematic director (Figure 5a), in agreement with observations reported in prior research.^[^ ^53^, ^54^ ^]^ The bending suggests contraction or shrinkage of the treated surface, which may be attributed to a slight reduction in mesogen alignment. Thermal actuation differs from the behavior observed in untreated samples (Figure 5b). After acid treatment, heating the material caused it to become flat and contract, although to a lesser extent than the 35% contraction observed before the treatment. Upon cooling, the strip curled longitudinally (Figure 5c). This behavior can be attributed to an anisotropic ordering of the mesogens across the thickness of the material, with reduced alignment on the acid‐treated side. Since the opposite surface of the film remains unaffected and retains its original degree of order, it expands more than the treated side upon cooling, thereby inducing the curling effect. As previously demonstrated for sufficiently thin films (thickness < 140 µm; ≈90 µm in our case), this change in alignment homogeneity may result from the selective protonation of one surface, leading to the formation of a bilayer‐like structure with a reduced average mesogen orientation on the treated side.^[^ ^55^ ^]^ Moreover, cleavage of the β‐aminoacrylate bonds under acidic conditions, which has been shown to occur within minutes under strongly acidic conditions,^[^ ^9^ ^]^ may tentatively contribute to the formation of a cross‐linking gradient across the film thickness, disrupting the uniform alignment. Upon illumination, untreated and acid‐treated films exhibit opposite contraction and expansion behaviors relative to each other, as shown in Supporting Information (Figure S23, Supporting Information).

Finally, the water‐responsive properties were evaluated by immersing the acid‐treated **LCE‐A20** strip in water and comparing its response to that of an untreated film (Figure 5d). In this case, the protonated amino groups can interact with the water molecules, enabling macroscopic deformation.^[^ ^56^ ^]^ Therefore, the hydrophilic surface undergoes isotropic swelling upon exposure to water, while the non‐treated surface remains hydrophobic and unresponsive to humidity. Since no changes in order occur on the non‐treated surface, the material bends around the nematic axis (along the direction perpendicular to the alignment direction),^[^ ^57^ ^]^ forming a tube with the swollen hydrophilic surface on the outer side, as shown in Figure 5e. After this initial swelling, the hydrated, acid‐treated strip can undergo a second‐stage actuation upon immersion in hot water (90 °C), recovering its original bent state toward the activated side, as shown and discussed in the Supporting Information (Figure S24, Supporting Information).

**Figure 6:**
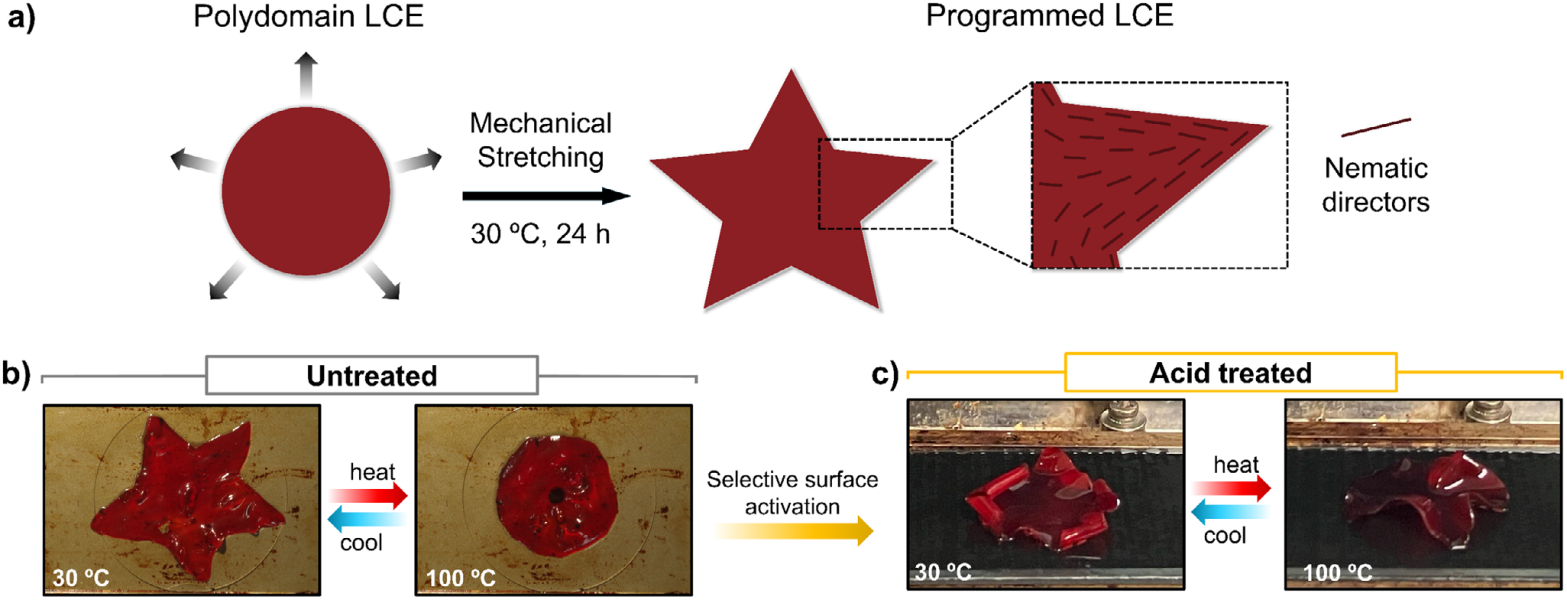
Programming complex orientation patterns: a star‐shaped soft actuator. a) Scheme of star‐shaped actuator preparation. b) Reversible shape change induced by thermal actuation before and c) after acid treatment (images correspond to the second heating‐cooling cycle).

As demonstrated, the ability to achieve acid‐activation of these materials at room temperature not only facilitates a straightforward response to water but also opens the potential for new functionalities when combined with responses to other stimuli, such as temperature or light. To prove the versatility of the present LCE system that arises from the combination of all the aforementioned features and its capacity to undergo dynamic bond exchange, a star‐shaped soft actuator was prepared as a proof of concept. Accordingly, a circular sample was symmetrically stretched along five arms (up to 100% of the length from the center of the circle to the outer edge of the circumference) and left undisturbed at 30 °C for 24 hours (**Figure** **6**a). After locking the alignment, the thermal cycle led to the reversible appearance of the circular shape upon heating (100 °C) and the recovery of the star shape at ambient temperatures (Figure 6b; Movie S1, Supporting Information), which arises from a gradual contraction of the outer arms while the central core remains flat upon heating (Figure S25, Supporting Information). Moreover, selective surface protonation of one of the star actuator's surfaces was accomplished (Figures S26 and S27, Supporting Information). Upon heating to 100 °C, a complex three‐dimensional circular structure emerges, while upon cooling, each arm of the star forms a curl as a result of the greater extension of the untreated surface than of the protonated one (Figure 6c), consistent with the mechanism described above for the rectangular strip. This behavior highlights the effectiveness of the treatment in inducing a predictable and multi‐responsive actuation to external stimuli for soft actuators with complex designs.

## 3 Conclusion

In this work, we developed a two‐stage strategy for LCE multi‐responsive actuators based on amino‐yne click chemistry at near ambient temperatures, through fast and spontaneous formation of β‐aminoacrylate cross‐links. Polydomain LCE materials exhibited tunable thermal and thermomechanical properties, dependent on the amount of dipropiolate cross‐linker and mesogenic monomers. After alignment programming via dynamic transesterification at 30 °C, the LCE actuators demonstrated reversible and reproducible thermal and light actuation behaviors, with maximum contraction around 35% of the original length, with a small influence of the cross‐linker amount. Tension developed under light illumination, however, increased with both cross‐linker and rigidity, with **LCE‐B20** exhibiting the highest tension across all light intensities and the fastest activation response. Despite this fact, the simple synthesis and still high performance of **LCE‐A20**, combined with its resistance to repeated activation cycles, make it the preferred material for further soft actuator development. Additionally, acid treatment on one surface made the material water‐sensitive, generating asymmetric hydrophilicity and disorder on the activated surface, therefore inducing distinct actuation behaviors if compared to those previously observed upon exposure to temperature and light in non‐acid‐treated samples.

Combined with the ability to perform dynamic bond exchange, this LCE formulation presents an opportunity to design structures with complex orientation patterns that respond to a variety of stimuli. Setting 30 °C as the working temperature for locking the alignment mitigates common issues associated with other dynamic actuators, such as undesired reactions that lead to network degradation when stimuli such as high temperatures are applied to induce the dynamic exchange. Additionally, using low temperatures prevents film contraction, thereby facilitating the handling of the material for the creation of more complicated designs. These features led to the development of a star‐shaped soft actuator as proof of concept, showcasing a variety of actuation behaviors. This amino‐yne approach thus demonstrates a pathway for the development of multi‐responsive LCE soft actuators, enabling the fabrication of materials with complex designs through a fast and spontaneous click reaction at near to room temperature. Importantly, the resulting β‐aminoacrylate bonds may offer pH‐sensitivity, as demonstrated in previous works for different polymeric networks, or dynamic behavior under specific conditions without the need of catalysts, opening the door to potential recyclability in future studies and applications.

## 4 Experimental Section

### Materials

1,6‐Hexanediol, propiolic acid, *para*‐toluenesulfonic acid, sodium azide, triphenylphosphine, 1,5,7‐triazabicyclo[4.4.0]dec‐5‐ene (TBD), and polyvinyl alcohol 4–88 were purchased from Sigma Aldrich. 2‐Methyl‐1,4‐phenylene bis(4‐((6‐(acryloyloxy)hexyl)oxy)benzoate) (**RM82**) was purchased from Synthon Chemicals. 1,5‐diaminopentane and 2‐[N‐ethyl‐4‐[(4‐nitrophenyl)diazenyl]anilino]ethyl prop‐2‐enoate also known as Disperse Red 1 acrylate (**DR1‐acr**) was purchased from Merck. The chemicals were used as received without further purifications. PVA solution (2.5 wt.%, H_2_O/EtOH (1/1)) was prepared by heating distilled water to 60 °C and adding the appropriate amount of PVA while stirring continuously. Once PVA was dissolved, the solution was cooled to room temperature, and the selected volume of ethanol was subsequently added. Synthetic procedures for dipropiolate cross‐linker, **RM‐diNH2,** and LCOs are reported in Supporting Information.

### Preparation of LCEs

**LCO‐A** or **B** (200 mg, 0.28 mmol of amine groups) and dipropiolate cross‐linker (10, 20, or 30 mol% in respect to amine groups) were separately dissolved in dichloromethane (0.7 and 0.3 mL for the LCO and cross‐linker, respectively) and sonicated until homogeneity at room temperature (15 min). After that, **DR1‐acr** (1 wt.%) and **nTBD** (1 wt.%) were dissolved in the cross‐linker solution adequately (additional information of the composition of the films is collected in Tables S2 and S3, Supporting Information). Both solutions were subsequently mixed, and the mixture was cast onto a spin‐coated PVA‐covered glass slide (24 × 50 mm). A polymeric film was obtained just after a few seconds. It was placed at room temperature overnight to obtain the dried LCE film. For material alignment, the film was detached from the glass by immersion in water and stretched to 100–150% at 30 °C for 24 h, followed by a thermal treatment of the free‐standing film at 100 °C for 3 min.

### Thermal Actuation

Materials were cut to have the longest side of the film corresponding to the alignment direction. Each LCE film was put on a silicone oil drop on the top of a glass plate, heated to 100 °C and the temperature stabilized for 2 min. LCE film's length was measured as *L_h_*. Subsequently, the material was cooled to 30 °C and, after 2 min, its length was measured again as *L_rt_*. All measurements were repeated in triplicate. Results are represented as function of the contraction (%) (mean ± SD (n = 3)):

(1) $$Contraction\left(\%\right)=\frac{L_{rt}-L_{h}}{L_{rt}}\times 100$$

Recovery (%) of the film's length from one heating‐cooling cycle (*n*) to the next one (*n+1*) is calculated using the following equation:

(2) $$Recovery\left(\%\right)=100-\frac{L_{rt\left(n\right)}-L_{rt\left(n+1\right)}}{L_{rt\left(n\right)}}\times 100$$

### Active Tension Characterization by Light Actuation

The force developed under irradiation by the LCEs were recorded in a custom‐made setup of a force transducer WPI SI‐KG4 connected to a force transducer amplifier SI‐BAM21‐LC and two LED lamps Thorlabs M470L5 emitting blue light (470 nm). The LEDs control and the force recording were effectuated by a LabVIEW program with a multichannel driver. The experiments were conducted in isometric conditions. LCE strips 2 mm × 1.5 cm (1.2 cm active part, between the clamps) were illuminated on both faces intermittently with frequency of 0.1 Hz and the selected illumination time.

### Acid Activation

For film activation, a 0.1 m HCl solution was poured into a Petri dish, and the LCE strip was left floating so that one of its surfaces remained permanently in contact with the acidic solution. The activation time was set to 3 minutes. Then, excess aqueous solution of the activated surface was gently dried with paper.

## Conflict of Interest

The authors declare no conflict of interest.

## Supporting information



Supporting Information

Supporting Information

## Data Availability

The data that support the findings of this study are available in the supplementary material of this article.
