# Supplementary material for: An Amino‐Yne Click Chemistry Approach for Multi‐Responsive Liquid Crystal Elastomer Actuators
Source: Small. 2025 Oct 28;21(49):e09070. doi: 10.1002/smll.202509070 (PMC12696798; doi:10.1002/smll.202509070)
Supplement: Supplementary file 1 — Supporting Information [file SMLL-21-e09070-s002.docx]

**Supporting Information**

An amino-yne click chemistry approach for multi-responsive liquid crystal elastomer actuators

Sara Bescós-Ramo,^1,2^ Marco Turriani,^3,4^ Camilla Parmeggiani,^3,5^ Milagros Piñol,^1,2^ Luis Oriol,^1,2*^ Daniele Martella^3,4,5*^

^1^Instituto de Nanociencia y Materiales de Aragón (INMA), CSIC-Universidad de Zaragoza, Zaragoza 50009, Spain

^2^Departamento de Química Orgánica, Facultad de Ciencias, Universidad de Zaragoza, 50009 Zaragoza, Spain

^3^European Laboratory for Non-Linear Spectroscopy (LENS) via N. Carrara 1, Sesto Fiorentino 50019, Italy

^4^Department of Physics and Astronomy, University of Florence, Via S. Sansone 1, Sesto Fiorentino 50019, Italy

^5^Department of Chemistry “Ugo Shiff”, University of Florence, Via della Lastruccia 3-13, Sesto Fiorentino 50019, Italy

* Corresponding authors:

E-mail address: loriol@unizar.es (L. Oriol); daniele.martella@unifi.it (D. Martella)

1. **Characterization techniques**

^1^H-NMR and ^13^C-NMR spectra were acquired on a Bruker Avance NEO 400 MHz spectrometer. The experiments were performed at room temperature in CDCl_3_. FT IR spectra were recorded on a Bruker Vertex 70 FT-IR spectrometer by preparing the samples on KBr pellets with a concentration of the product of 1-2% (w/w), or ATR (PerkinElmer Spectrum Two). Thermal characterization was performed by Differential Scanning Calorimetry (DSC) using a Q-2000 DSC from TA Instruments at a 10 °C min^−1^ scanning rate for **RM-diN_3_** and **RM-diNH_2_** or 20 °C min^−1^ for all other materials. Polarized Optical Microscopy (POM) was performed on a Zeiss Axio Observer A1 equipped with a Linkam PE120 hot stage and an Axio camera. Dynamic Mechanical Analysis (DMA) was recorded on a PerkinElmer DMA 8000 in the shear mode and with rectangular samples mounted on the clamp. Tensile tests were performed with the Modular Force Stage by Linkam Scientific Instrument Ltd. Strips of LCE were cut from larger samples, secured in between the clamps, and stretched with the mobile crosshead moving at a constant speed of 20 micron s−1. The force and the displacement of the mobile crosshead were recorded allowing to calculate the engineering stress (σ = force/initial specimen cross-section) and strain (ɛ = displacement/initial film gauge length). Gel content tests were performed by immersing LCE films (*W_0_*) into toluene at room temperature for 72 h. Solvent was renewed every 24 h. After that, the sample was dried under vacuum until its weight reached a constant value (*W_f_*). The gel fraction was calculated following equation: $Gel content=\frac{W_{f}}{W_{0}}\times100$.

1. **Synthesis description, molecule characterization and preparation procedures**
   1. **Dipropiolate cross-linker and liquid crystal monomers**

**Scheme S1. Synthetic scheme of dipropiolate cross-linker.

*Synthesis of dipropiolate cross-linker.* 1,6-Hexanediol (3 g, 25.38 mmol) was dissolved in toluene (150 mL). Subsequently, propiolic acid (5.70 g, 76.16 mmol) and *para*-toluenesulfonic acid (483 mg, 2.54 mmol) were added to the solution. The mixture was subjected to reflux for 18 h using a Dean-Stark apparatus to facilitate the removal of water and promote the reaction towards completion. The reaction was cooled to room temperature and then the solvent removed under vacuum. The residue was re-dissolved in dichloromethane (DCM) (200 mL) and washed with a saturated solution of NaHCO_3_ (3×60 mL), H_2_O (60 mL) and a saturated solution of NaCl (60 mL). The organic phase was dried over MgSO_4_, filtered and evaporated to dryness. The resultant product was purified by flash column chromatography on silica gel, initially eluting with 95:5 hexane/ethyl acetate, gradually increasing the polarity to finish with a 9:1 mixture (R_f_=0.3). The target compound was obtained as a white powder. Yield: 4.4 g, 79 %. ^1^H NMR (400 MHz, CDCl_3_, ppm): *δ*=4.20 (d, *J* = 6.6 Hz, 4H), 2.87 (s, 2H), 1.75-1.66 (m, 4H), 1.44-1.39 (m, 4H). ^13^C NMR (100 MHz, CDCl_3,_ ppm): *δ*=152.9, 74.9, 74.7, 66.3, 28.3, 25.5. FT IR (ATR, cm^-1^): υ=3225, 2986-2857, 2110, 1697.


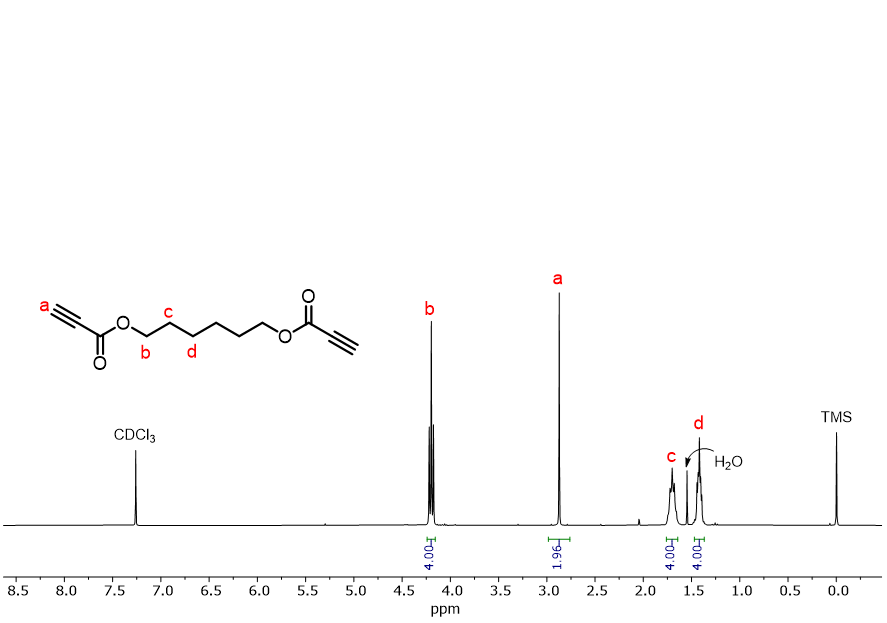


Figure S1. ^1^H NMR (400 MHz, CDCl_3_, ppm) spectrum of dipropiolate cross-linker.


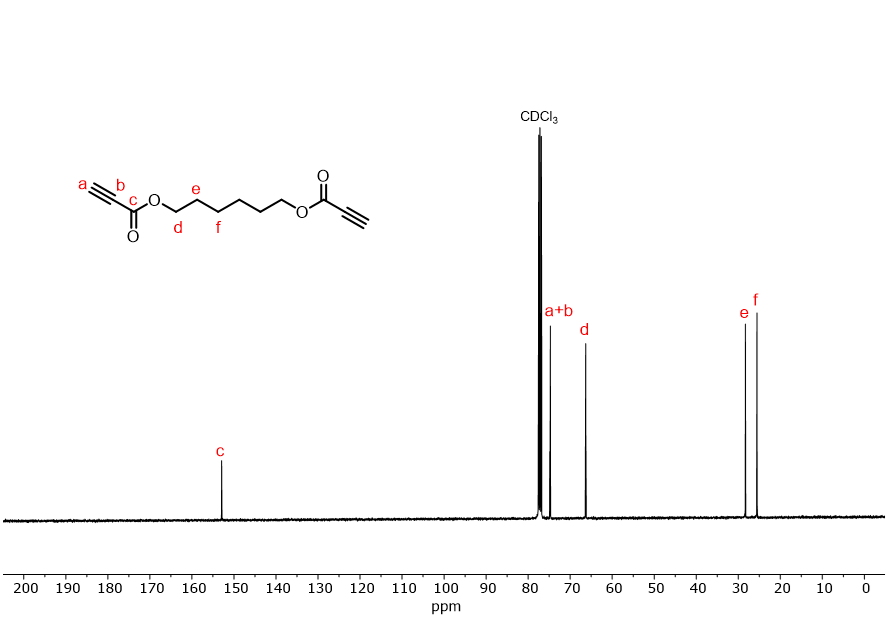


Figure S2. ^13^C NMR (100 MHz, CDCl_3_, ppm) spectrum of dipropiolate cross-linker.

Scheme S2. Synthetic scheme of **RM-diNH_2_.**

*Synthesis of 2-methyl-1,4-phenylene bis(4-((6-azidohexyl)oxy)benzoate) (****RM-diN_3_****).* 2-Methyl-1,4-phenylene bis(4-((6-bromohexyl)oxy)benzoate) was synthetized following a previously reported procedure.^[1]^ The aforementioned product (3.8 g, 5.50 mmol) was dissolved in anhydrous *N*,*N*-dimethylformamide (DMF) (50 mL) and degassed with argon. To this solution, sodium azide (2.15 g, 33 mmol) was added and the reaction was stirred under argon at 40 ºC for 18 h. Then, water (100 mL) was added and extracted with a mixture of hexane/ethyl acetate (1:1) (3×100 mL). The organic extracts were combined and washed with water (100 mL) and a saturated solution of NaCl (100 mL). Then, the organic layer was dried over MgSO_4_, filtered and concentrated under vacuum. The residue was purified by flash column chromatography on silica gel, eluting with a 9:1 hexane/ethyl acetate mixture and gradually increasing the polarity to finish with 8:2 hexane/ethyl acetate (R_f_=0.45) to afford the target product as a white solid. Yield: 2.5 g, 71 %.^1^H NMR (400 MHz, CDCl_3,_ ppm): δ=8.18–8.12 (4H, m), 7.19-7.07 (3H, m), 7.00-6.95 (4H, m), 4.08–4.04 (4H, m), 3.30 (4H, t, *J* = 6.8 Hz), 2.24 (3H, s), 1.89-1.81 (4H, m), 1.69-1.62 (4H, m), 1.58-1.43 (8H, m). ^13^C NMR (100 MHz, CDCl_3,_ ppm): δ=165.1, 164.7, 163.6 (2C), 148.6, 147.2, 132.4 (4C), 131.9, 124.3, 123.0, 121.8, 121.6, 120.2, 114.5 (2C), 114.4 (2C), 68.2 (2C), 51.5 (2C), 29.1 (2C), 28.9 (2C), 26.6 (2C), 25.8 (2C), 16.6. FTIR (KBr, cm^-1^): υ=2943, 2866, 2097, 1731, 1606.


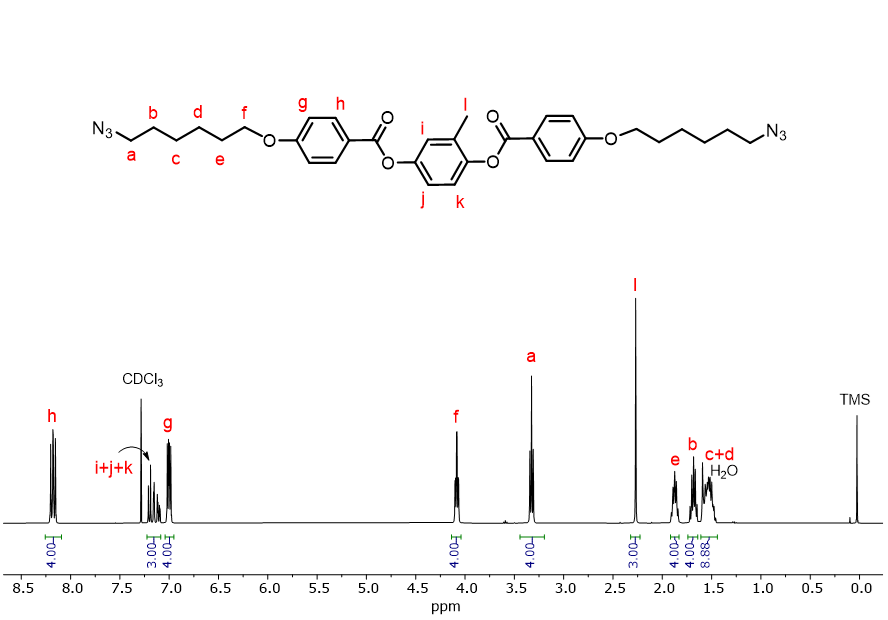


Figure S3. ^1^H NMR (400 MHz, CDCl_3_, ppm) spectrum of **RM-diN_3._**


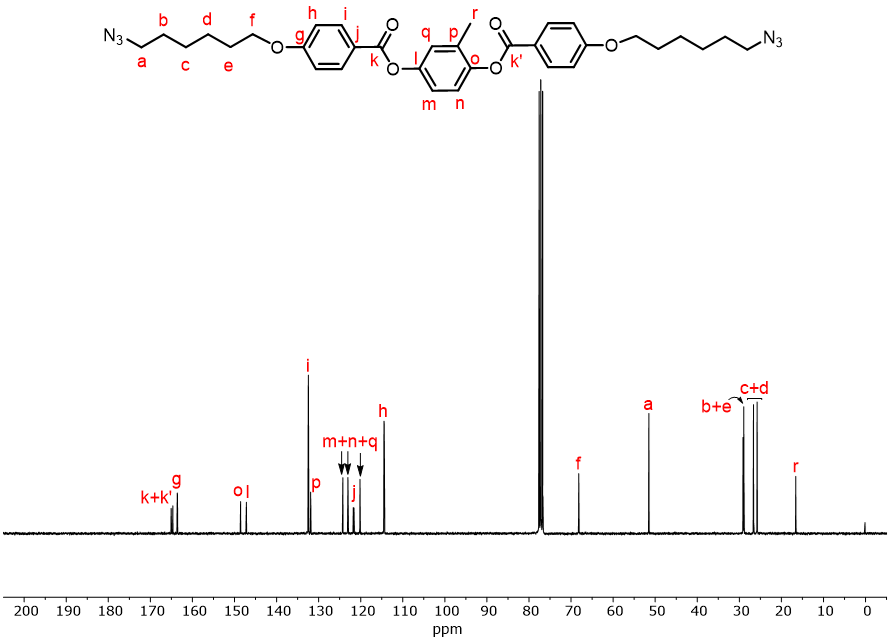


Figure S4. ^13^C NMR (100 MHz, CDCl_3_, ppm) spectrum of **RM-diN_3_**.


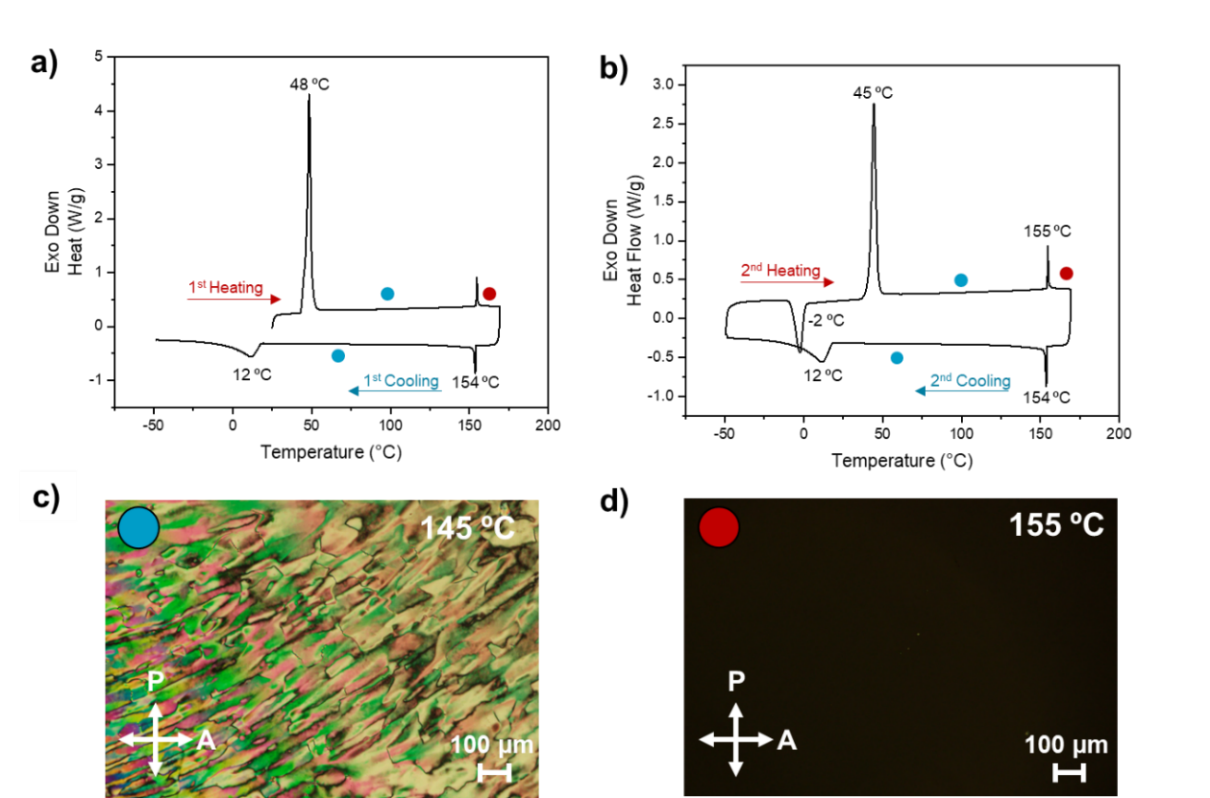


Figure S5. DSC curves recorded at 10 °C/min corresponding to a) the first heating and b) the second cooling of the precursor **RM-diN_3_**. POM images of c) the nematic phase at 145°C on cooling and d) isotropic phase.

*Synthesis of 2-methyl-1,4-phenylene bis(4-((6-aminohexyl)oxy)benzoate) (****RM-diNH_2_****).* **RM-diN_3_** (1.2 g, 1.95 mmol) was dissolved in anhydrous tetrahydrofuran (THF) (20 mL) and degassed with argon. Triphenylphosphine (1.23 g, 4.69 mmol) was subsequently solved in THF (5 mL) and added dropwise to the reaction mixture, which was stirred under argon at 40 ºC for 8 h. The flask was cooled in an ice bath and water (6 mL) was added dropwise after which the reaction was left stirring overnight. Solvent was evaporated under vacuum and by lyophilization. The solid was repeatedly washed with a cold mixture of hexane/ethyl acetate (7:3) to afford the target product as a white solid. The product must be stored at low temperature under an argon atmosphere Yield: 603 mg, 55 %.^1^H NMR (400 MHz, CDCl_3,_ ppm): δ=8.17–8.12 (4H, m), 7.19-7.07 (3H, m), 6.99-6.96 (4H, m), 4.07–4.03 (4H, m), 2.72 (4H, t, *J* = 6.9 Hz), 2.24 (3H, s), 1.87-1.81 (4H, m), 1.54-1.38 (12H, m). ^13^C NMR (100 MHz, CDCl_3,_ ppm): δ=165.0, 164.6, 163.6, 163.5, 148.4, 147.0, 132.3 (2C), 132.3 (2C), 131.8, 124.1, 122.9, 121.6, 121.4, 120.1, 114.4 (2C), 114.3 (2C), 68.2, 68.2, 42.2 (2C), 33.8 (2C), 29.1 (2C), 26.7 (2C), 25.9 (2C), 16.46. FT IR (KBr, cm^-1^): υ=3332, 2939, 2860, 1726, 1606.


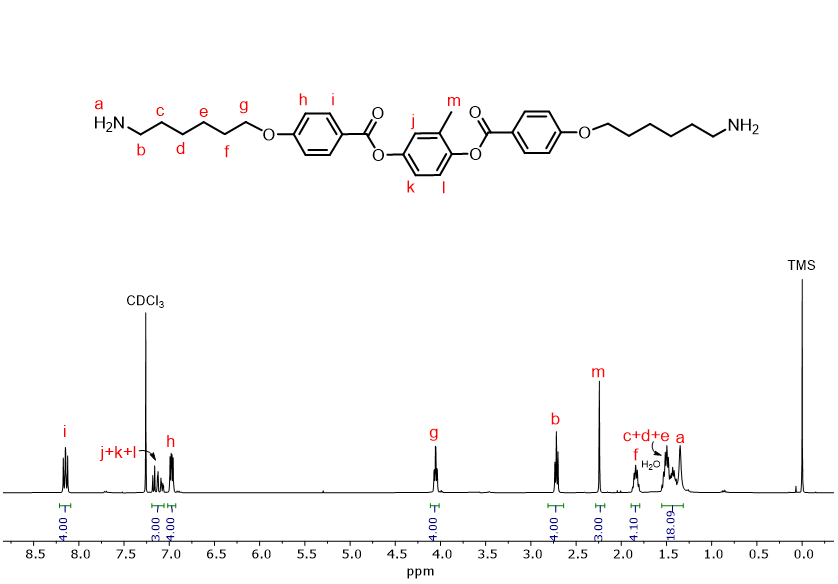


Figure S6. ^1^H NMR (400 MHz, CDCl_3_, ppm) spectrum of **RM-diNH_2_**.


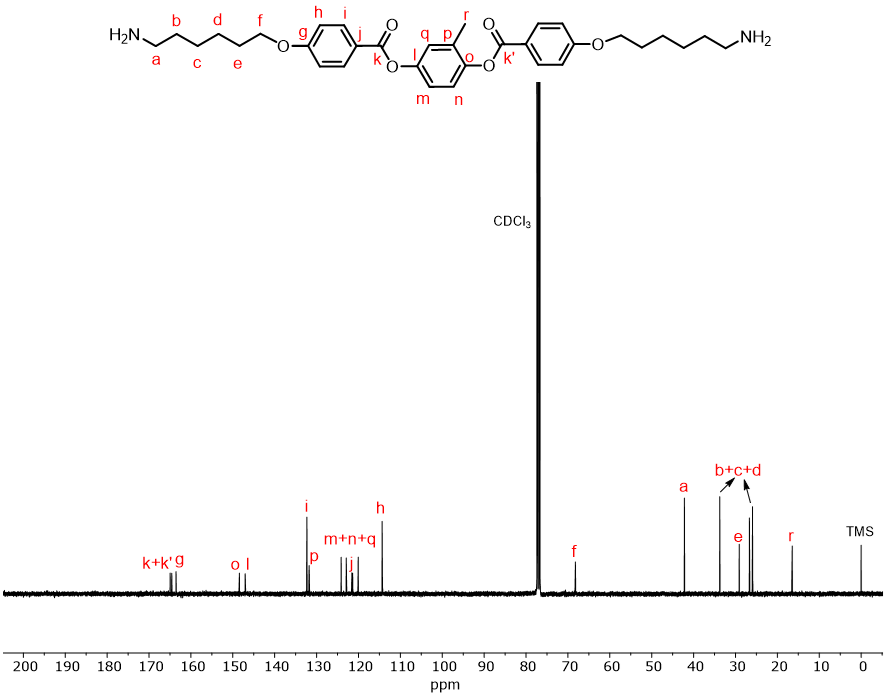


Figure S7. ^13^C NMR (100 MHz, CDCl_3_, ppm) spectrum of **RM-diNH_2_**.


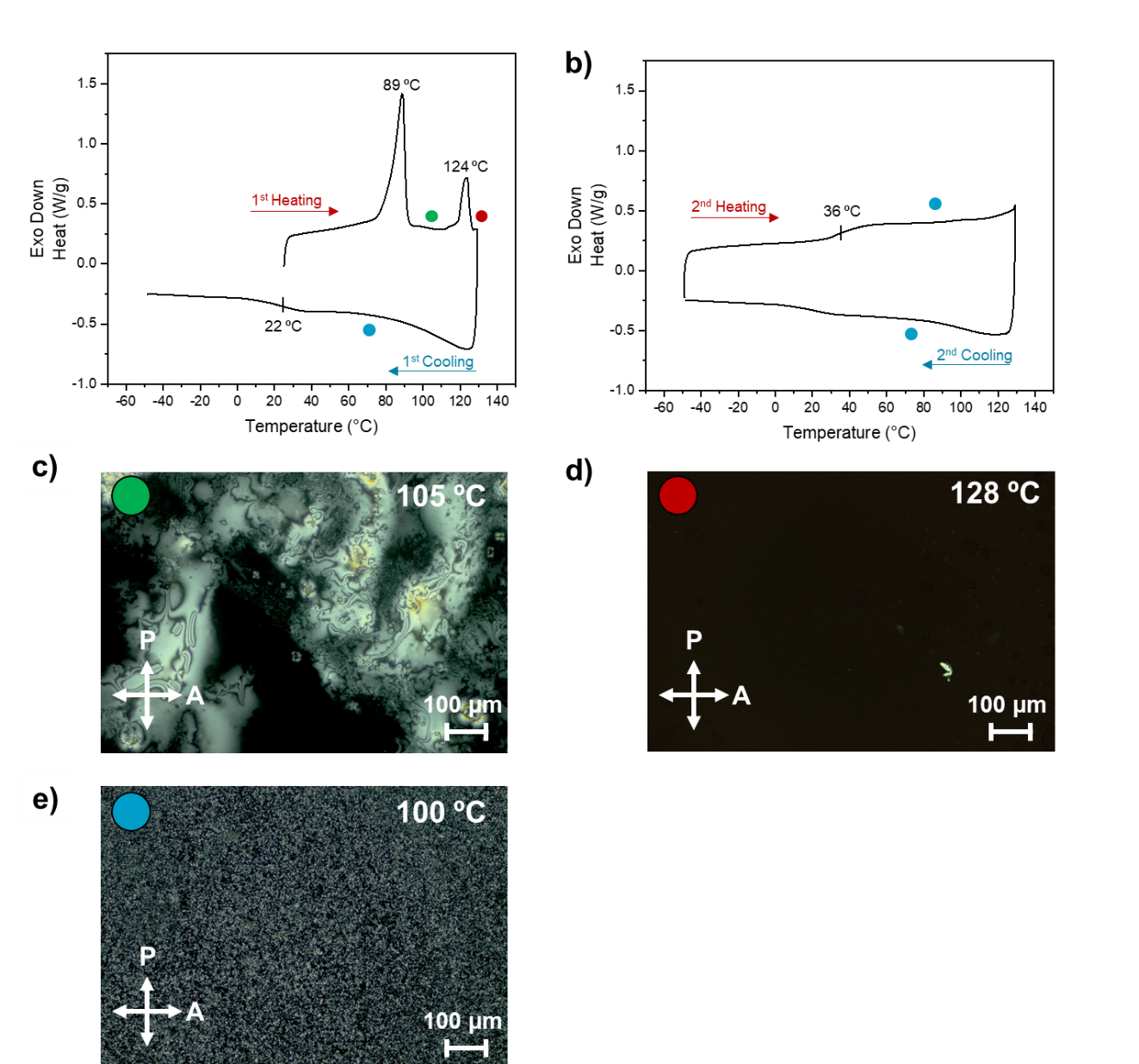


Figure S8. DSC curves recorded at 10 °C/min corresponding to a) the first and b) second cycles of heating and cooling of **RM-diNH_2_**. POM images of c) the nematic phase on the first heating at 105°C, d) the isotropic phase at 128 °C after the first heating ramp and e) the nematic phase on cooling at 100 ºC. After the first heating cycle, no crystallization is observed during either cooling or subsequent heating. Moreover, the textures observed by POM before and after reaching the isotropic phase in the first heating cycle, together with what is observed in the second DSC curve, suggest that structural changes are taking place, most likely due to degradation or side reactions involving the amine groups.

- 1. **Liquid Crystal Oligomers**

*Synthesis of* ***LCO-A****.* RM82 (1 g, 1.49 mmol) and 1,5-diaminopentane (195 µL, 1.63 mmol) were dissolved in DCM (3 mL). The solution was stirred at room temperature for 36 h, after which the solvent was removed under vacuum. **LCO-A** was obtained as a white viscous liquid. Yield: 92 %.

Figure S9. ^1^H NMR (400 MHz, CDCl_3_, ppm) spectrum of **LCO-A**.

*Synthesis of* ***LCO-B****.* **RM-diNH_2_** (325 mg, 0.58 mmol) and 1,5-diaminopentane (69 µL, 0.58 mmol) were dissolved in DMF (0.5 mL). A solution of RM82 (710 mg, 1.05 mmol) in a DMF/DCM (1:1) (2.5 mL) was added to the reaction flask. The solution was stirred at room temperature for 36 h, after which DCM was removed under vacuum at room temperature. The resultant solution was precipitated into diethyl ether (50 mL). The polymer was isolated by decantation and dried under vacuum yielding **LCO-B** as a yellowish viscous liquid. Yield: 81 %.


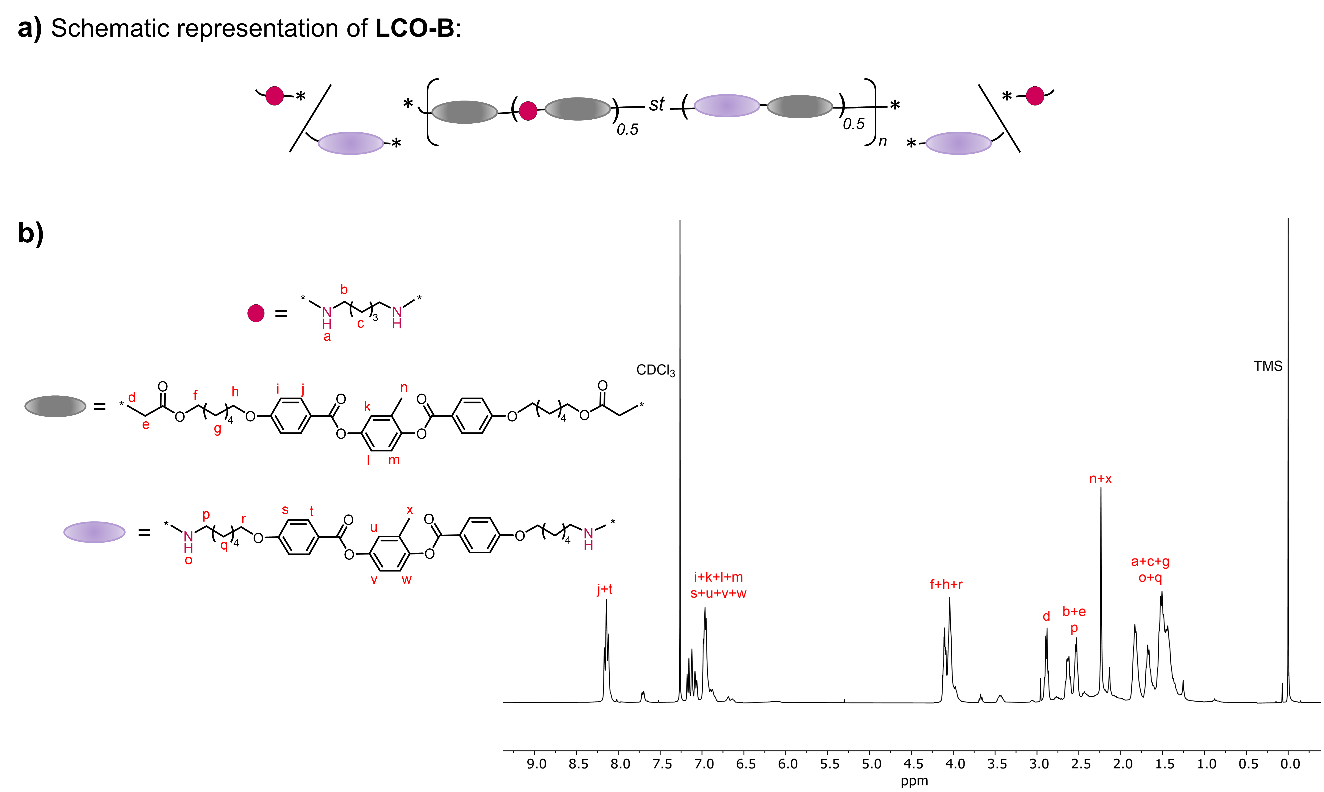


Figure S10. A) Schematic representation of **LCO-B** and b) ^1^H NMR (400 MHz, CDCl_3_, δ (ppm)) spectrum of **LCO-B**.

The theoretical degree of polymerization (DP_theor_) can be estimated using Carothers equation^[2]^: $DP_{theor}=\frac{1}{1-p}$ Equation S1

Where *p* refers to the fractional monomer conversion, defined as:

$p=\frac{N_{0}-N}{N_{0}}$ Equation S2

*N_0_* is the number of molecules present initially and *N* is the number of unreacted molecules after time *t*. ⁠Since complete conversion of acrylate monomer was checked by ^1^H NMR analysis, *N* refers to the excess in amine monomers, and the Carothers can be simplified:

$DP_{\boldsymbol{LCO-B}, theoretical}=\frac{No}{N}$ Equation S3

The experimental degree of polymerization (DP_exp_) was estimated for **LCO-A** through Equation S4, by relating the integration of the end-group protons (Figure S7, *C****H_2_***NH_2_, 2.69 ppm, 4H, labelled as ***b***) with the integration of the aromatic protons of the mesogenic unit (Figure S7, 8.14 ppm, 4H per repetitive unit, labelled as ***l***). These signals were selected because they appear in distinct and well-separated regions of the spectrum.

${DP}_{exp}=\frac{Integration of \boldsymbol{l} protons}{Integration of \boldsymbol{b}protons}$ Equation S4

Table S1. Theoretical and experimental DP for both LCOs. Data from N_0_ and N is collected from the corresponding synthetic procedures.

| **Compound** | **N_0_ (mmol)** | **N (mmol)** | **DP_theor_** | **DP_exp_** |
| --- | --- | --- | --- | --- |
| **LCO-A** | 1.63 | 0.14 | 11 | 10 |
| **LCO-B** | 1.16 | 0.11 | 10 | - |

Figure S11. Scheme of minor side-reactions of a) Aza-Michael addition of secondary amines and b) amidation reaction between excess of primary amines and oligomer esters. c) Expanded ^1^H NMR and d) homonuclear correlation spectroscopy (COSY) spectrum of **LCO-A** showing characteristic signals the aforementioned side-reactions products.


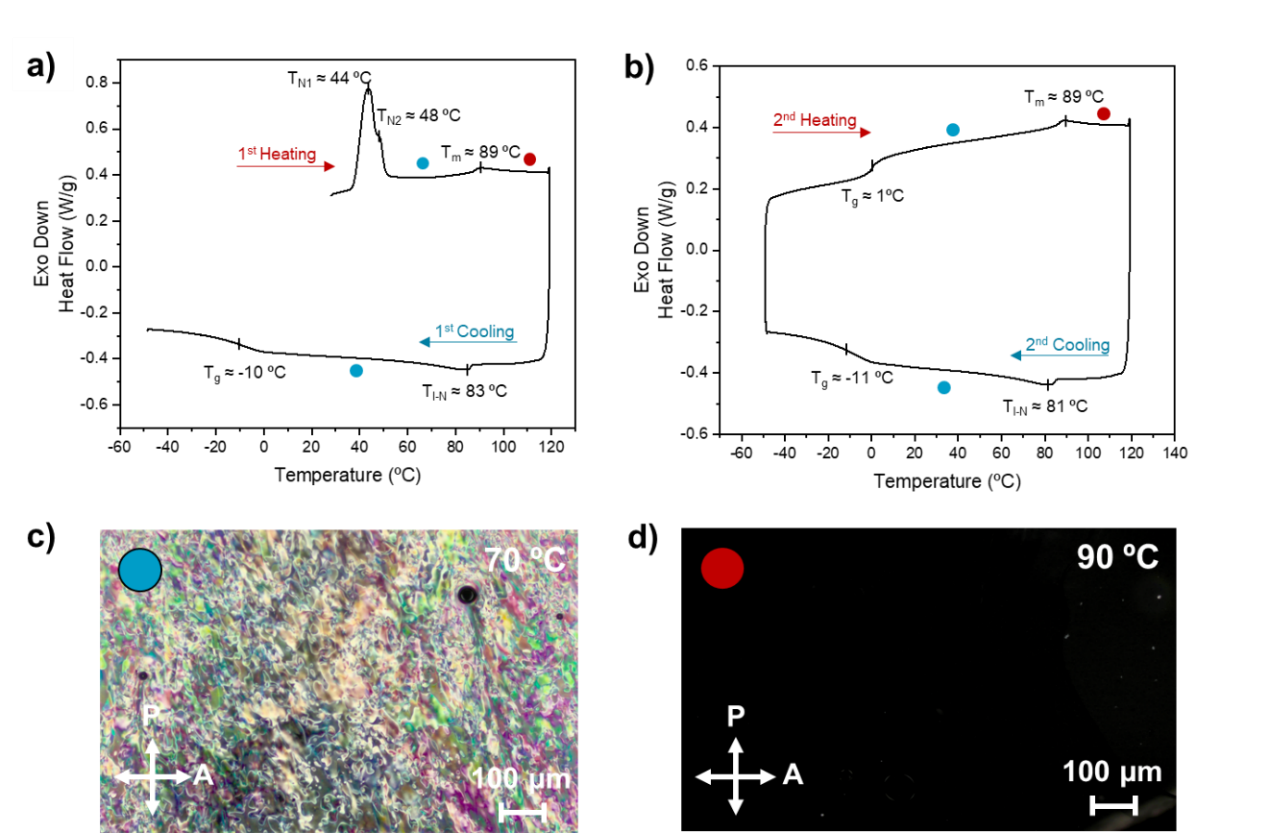


Figure S12. DSC curves recorded at 10 °C/min corresponding to a) the first and b) second cycles of heating and cooling of **LCO-A**. POM images of c) the nematic phase at 70°C on heating and d) the isotropic phase at 90 °C on heating.


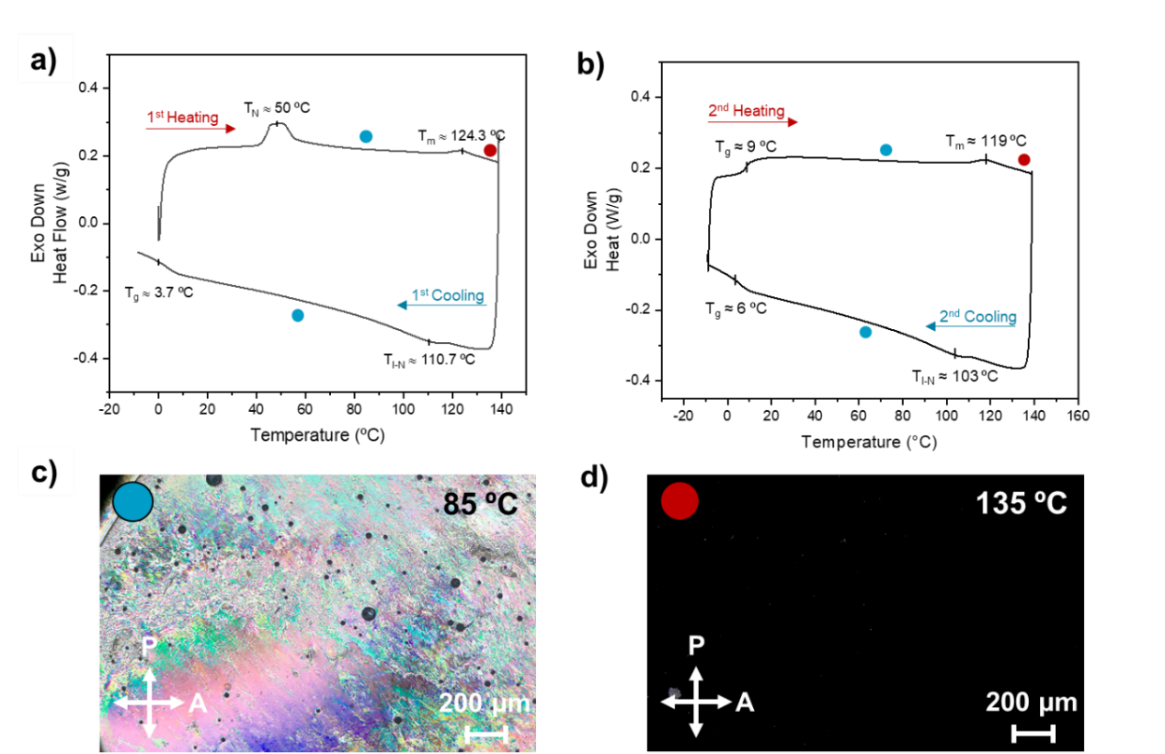


Figure S13. DSC curves recorded at 10 °C/min corresponding to a) the first and b) second cycles of heating and cooling of **LCO-B**. POM images of c) the nematic phase at 85°C on heating and d) the isotropic phase at 135 °C on heating.

- 1. **Liquid Crystal Elastomer Films**

*Synthesis of* ***nTBD****.* **nTBD** synthesis was carried out following a previously described procedure.^[3]^ 1,5,7-Triazabicyclo[4.4.0]dec-5-ene (TBD) (1 g, 7.18 mmol) was dissolved in ethanol (5 mL), and acetic acid (410 µL, 7.18 mmol) was added dropwise. The mixture was stirred at room temperature for 24 h and the solvent removed under reduced pressure at 60 ºC, yielding **nTBD** as a white solid. Yield: 97 %.

*Composition of LCE films.* The composition of the LCOs for preparing the LCE films are provided in Table S2, adjusted to yield 200 mg of each LCO, which were subsequently used for film preparation. Table S3 reports the amount of each reactant used for the preparation of the films,

Table S2. Composition of the LCOs for LCE films preparation. The data in the table include the amounts to obtain 200 mg of corresponding oligomer.

| **LCO** | **RM82 (mmol)** | **1,5-diaminopentane (mmol)** | **RM-diNH2 (mmol)** |
| --- | --- | --- | --- |
| **LCO-A** | 0.25 | 0.28 | - |
| **LCO-B** | 0.25 | 0.14 | 0.14 |

Table S3. Composition of the LCE films. The data in the table include the quantities used for the preparation of the LCE films.

| **LCE film** | **LCO-A (mmol)^[a]^** | **LCO-B (mmol)^[a]^** | **Dipropiolate cross-linker (mmol)** | **DR1-acr (wt %)^[b]^** | **nTBD (wt %)^[b]^** |
| --- | --- | --- | --- | --- | --- |
| **LCE-A10** | 0.28 | - | 0.028 | 1 | 1 |
| **LCE-A20** | 0.28 | - | 0.056 | 1 | 1 |
| **LCE-A30** | 0.28 | - | 0.084 | 1 | 1 |
| **LCE-B20** | - | 0.28 | 0.056 | 1 | 1 |

^[a]^: mmol of free amino groups, according to the amount of amine monomer used in the synthesis of each LCO.

^[b]^: The weight percentage (wt %) was calculated relative to the combined final mass of the LCO and the dipropiolate cross-linker.

Table S4. Thermal properties of LCMs and LCOs.

| **Compound** | **Phase transition temperatures (ºC)** | **T_g_^[d]^ (ºC)** |
| --- | --- | --- |
| **RM-diN3^[a]^** | h: Cr1 -2 Cr2 45 N 155 (1.9) I  c: I 154 (1.8) N 12 Cr | **-** |
| **RM-diNH2^[b]^** | h: Cr 89 N 124 (7.5) I  c: I 117^[c]^ N 22 g | - |
| **LCO-A^[a]^** | h: g 1 N 89 I  c: I 81 N -11 g | 1 |
| **LCO-B^[a]^** | h: g 9 N 119 I  c: I 103 N 6 g | 9 |

^[a][b]^: Transition temperatures and enthalpies were determined by DSC from the onset of the transition obtained during the second^[a]^ or first ^[b]^ heating (h) and cooling (c) cycles (10 ºC min^-1^). Within parentheses is reported the enthalpy of the transition (kJ mol^-1^) determined from the integration of the peak.

^[c]^: Data determined from POM images although clear decomposition is observed after heating above approximately 130 ºC.

^[d]^: Determined from the midpoint of the baseline jump during the second heating cycle.

1. **Thermomechanical and tensile characterization**


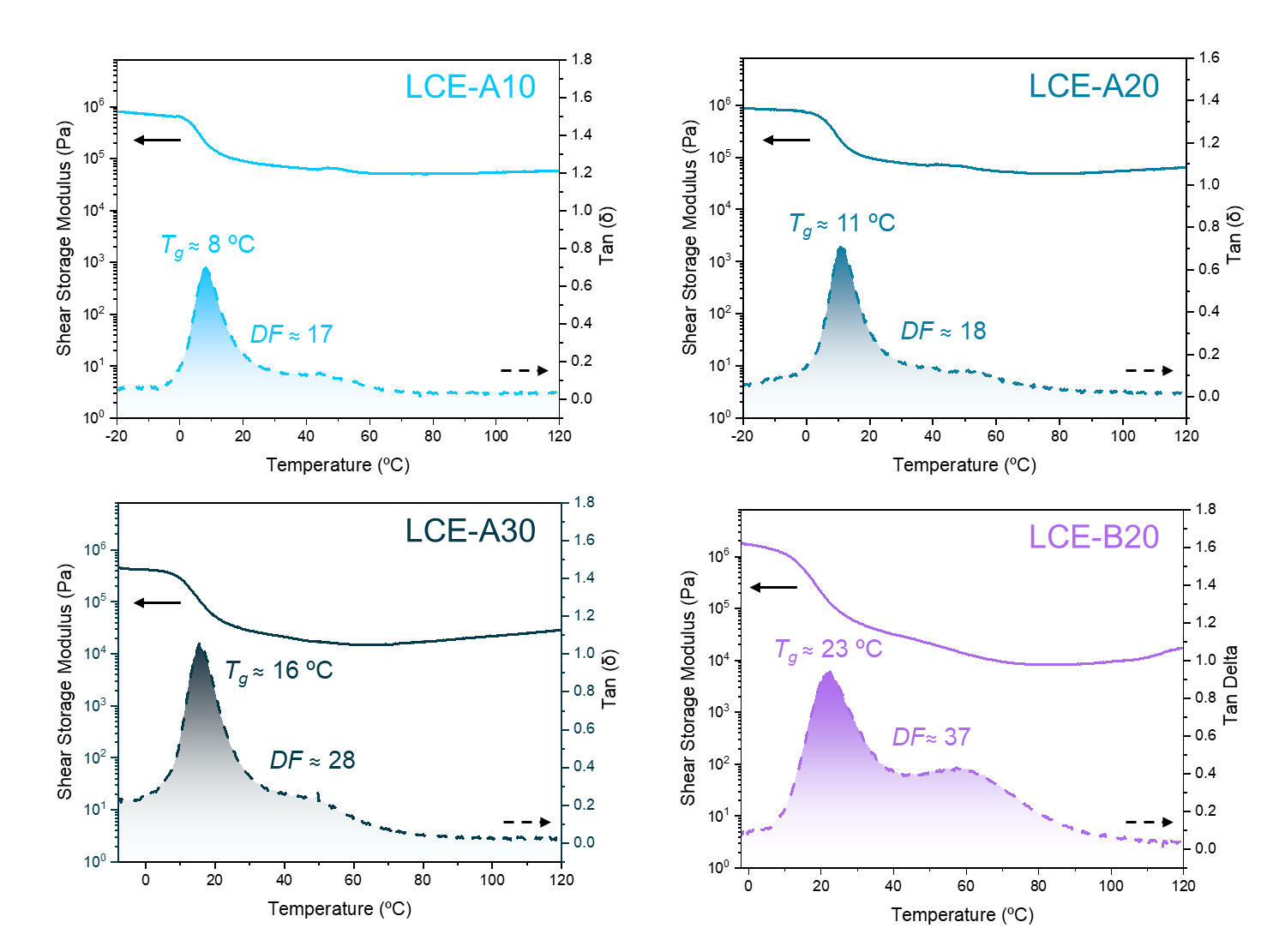


Figure S14. DMA experiments representing shear storage modulus-temperature (solid lines) and tan (𝛿)-temperature (dashed lines) curves of the LCE samples (temperature ramp rate = 2 °C/min, frequency = 1 Hz, and parallel plate geometry). T_g,DMA_ was determined as the temperature corresponding to the maximum of tan δ curves. The dissipation factor (DF) was determined as the area under the tan δ curve between T_g_ – 30 ºC (for **LCE-A20**) or T_g_ – 20 ºC (for **LCE-B20**) and 120 ºC (scan rate = 2 °C/min, frequency = 1 Hz, and parallel plate geometry).


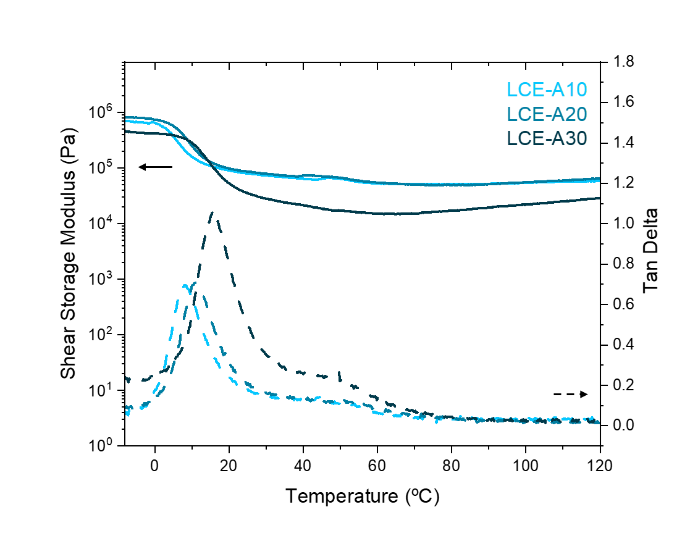


Figure S15. DMA experiments representing shear storage modulus-temperature (solid lines) and tan (𝛿)-temperature (dashed lines) curves, showing the comparison of **LCE-A10/20/30** samples (temperature ramp rate = 2 °C/min, frequency = 1 Hz, and parallel plate geometry).





Figure S16. Tensile characterization. Representative stress-strain curves of polydomain LCE-A20 and LCE-A30 films.

Table S5. Mechanical parameters calculated from the stress-strain curves. Tests were done in triplicate and average mechanical properties were calculated, namely Young's modulus (E), stress at break (σ_B_) and strain at break (ɛ_B_).

|  | **E [MPa]** | **σ_B_ [MPa]** | **ɛ_B_ [%]** |
| --- | --- | --- | --- |
| **LCE-A20** | 4.9 0.5 | 4.2 0.5 | 179 23 |
| **LCE-B20** | 11.2 0.6 | 9.1 0.4 | 126 19 |

1. **Thermal actuation**


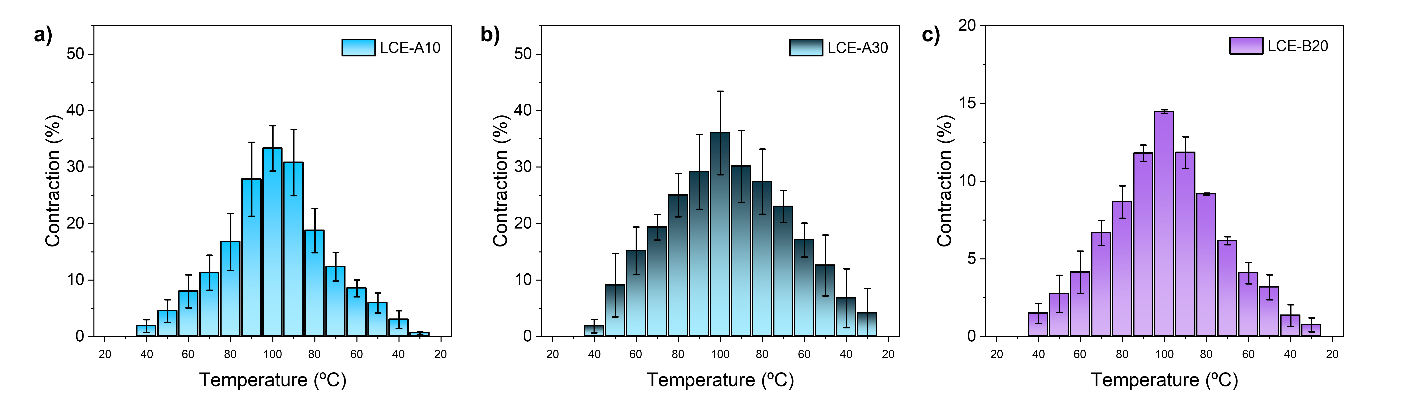


Figure S17. Reversible contraction (%) during the first thermal actuation test (from 30 to 100 °C and back) for a) **LCE-A10**, b) **LCE-A30** and c) **LCE-B20.**


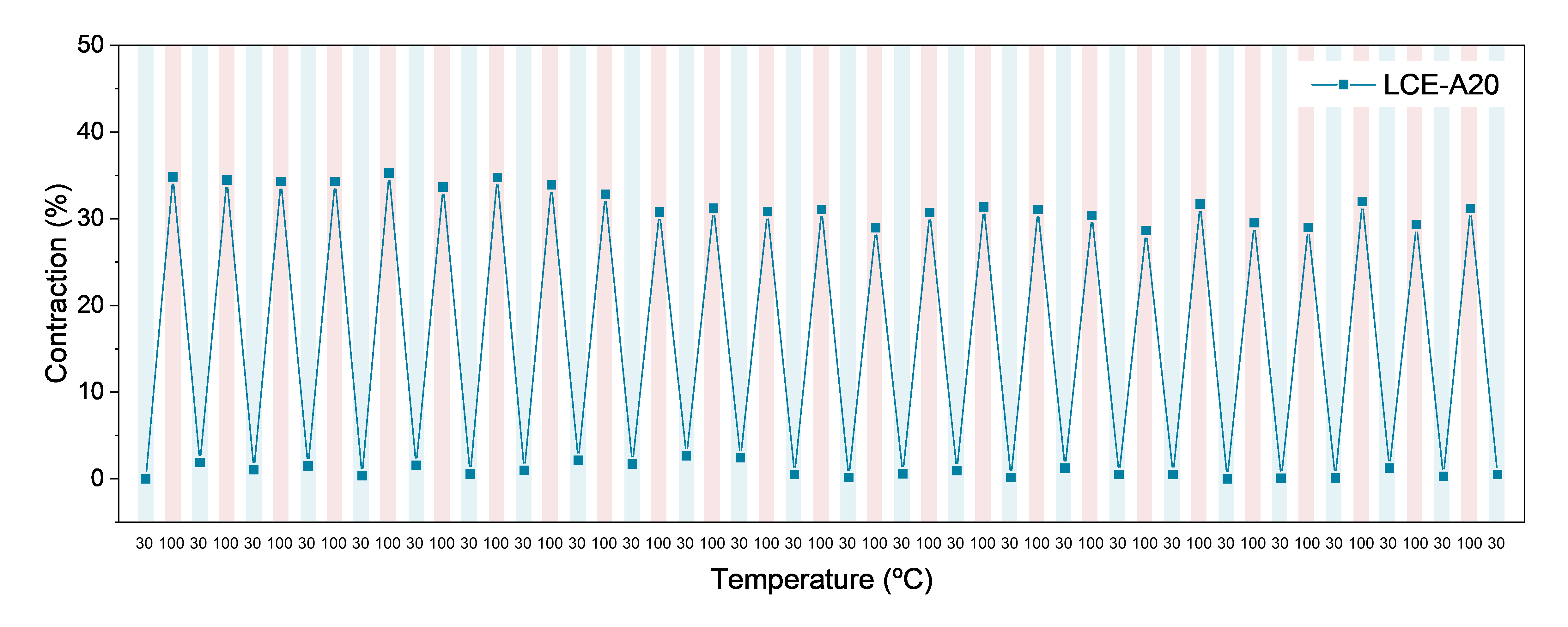


Figure S18. 25 thermal actuation cycles of **LCE-A20** between 30 and 100 °C.

Figure S19. Maximum contraction (%) of **LCE-A20** as function of stretch holding time.

1. **Light actuation**


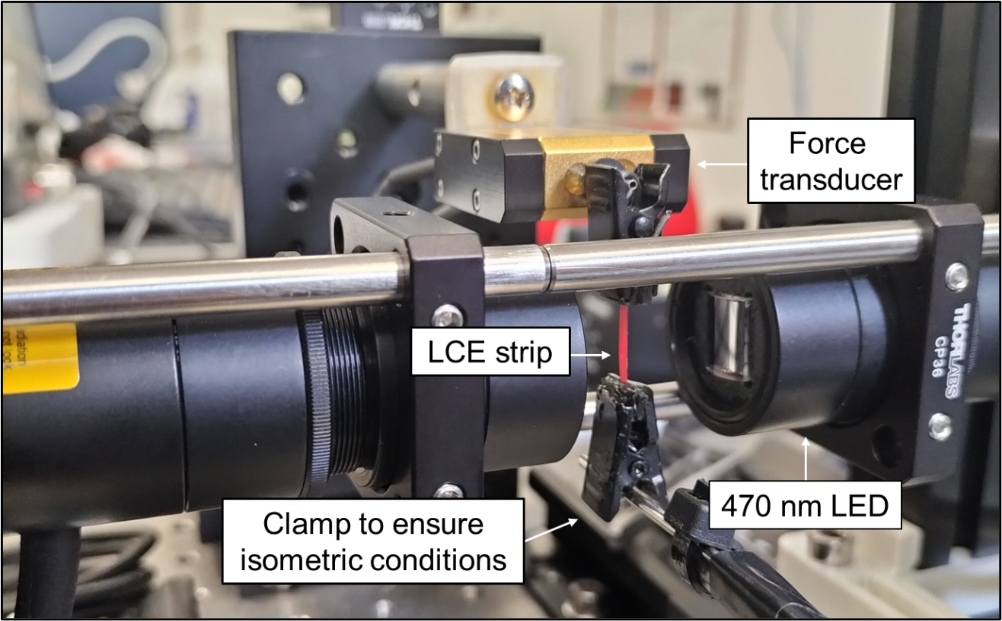


Figure S20. Image showing the setup for force measurement.


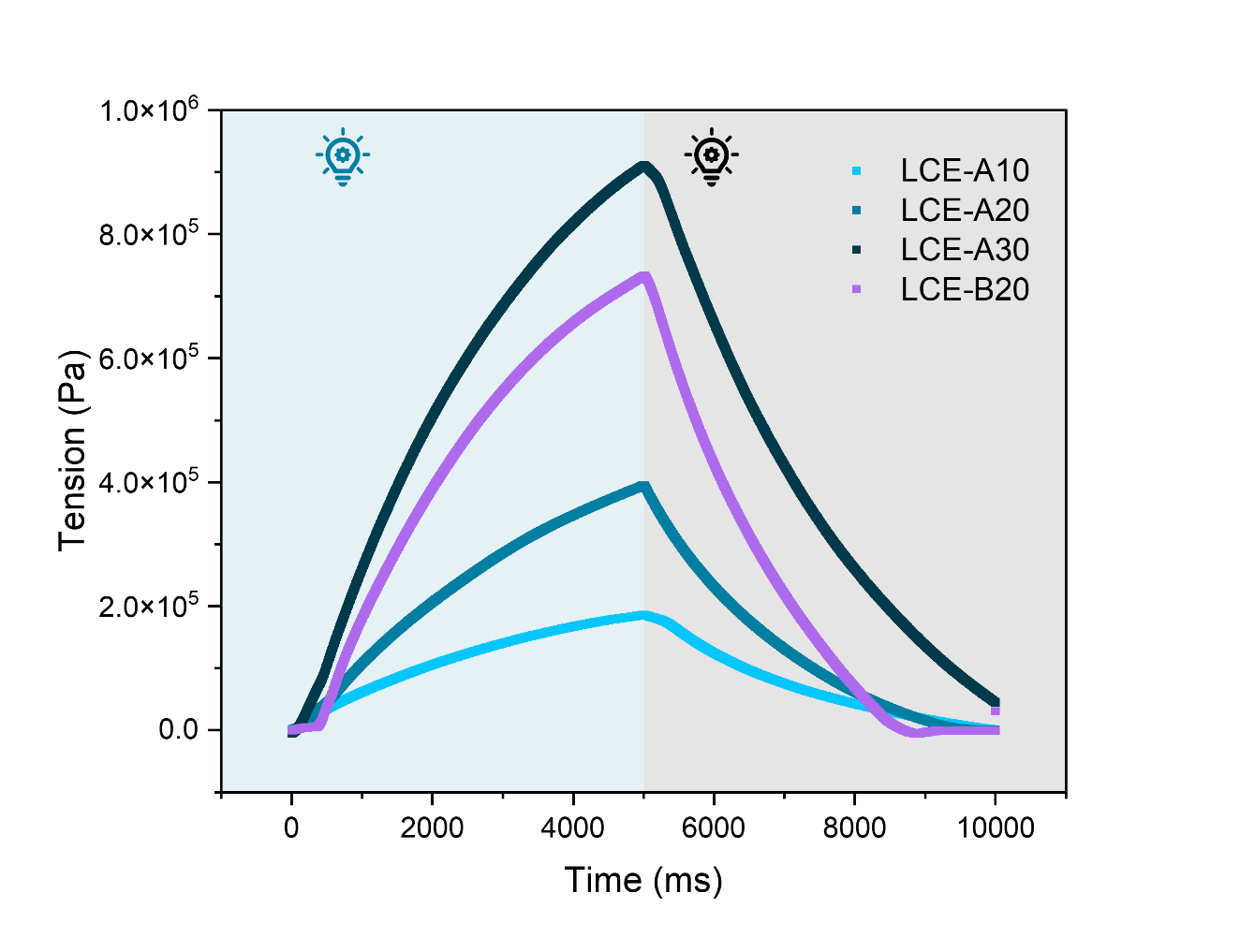


Figure S21. Mean trace of activation and relaxation cycle for each material under pulsed irradiation (6.2 mW/mm^2^).

1. **Acid activation**

**
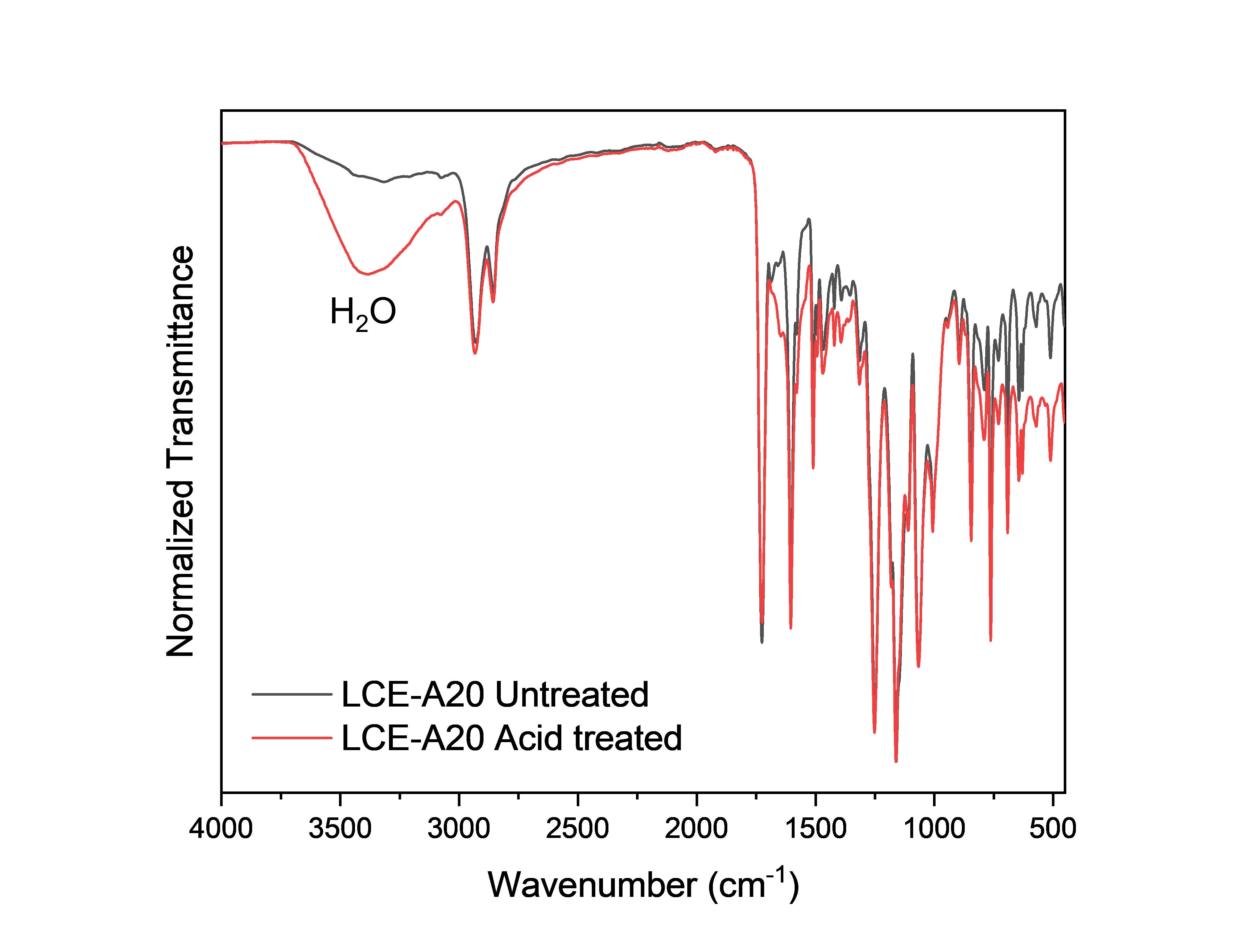
**

Figure S22. ATR of **LCE-A20** before (black line) and after (red line) acid treatment.

1. **Actuation performance after acid activation**

*Thermal actuation after one-surface acid activation.* For this experiment, activation time was increased up to 5 min in order to obtain a more curled strip, which made the thermal actuation more evident. After drying, protonated strip was put on a silicone oil drop on the top of a glass plate and heated from 30 ºC to 100 ºC and back, stabilizing the temperature for 2 min at each temperature.

*Light actuation after one-surface acid activation.* The strip was mounted vertically, with only the upper part attached to a clamp, and the protonated side positioned 10 cm from a blue light lamp emitting at 𝜆 = 450 nm. The photo with blue light on was taken after 5 s of continuous illumination.

*Water responsiveness after one-surface acid activation.* The strip was immersed in distillated water for 30 s.


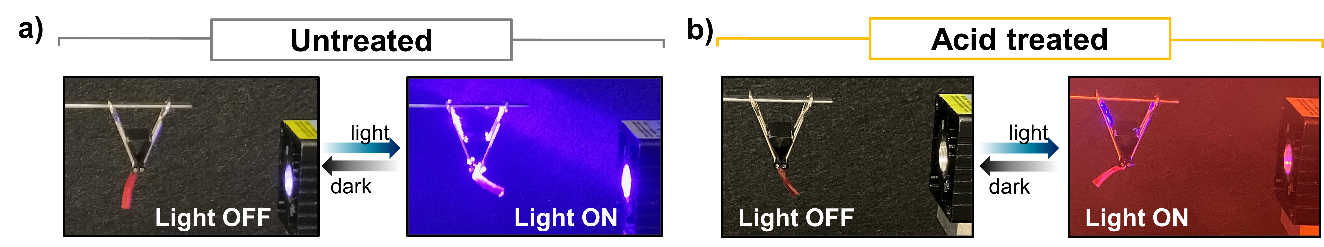


Figure S23. Reversible light actuation after illuminating the a) untreated and b) acid treated film, for which the protonated surface is facing the light source (an orange filter was used to facilitate visualization while illumination with blue light). Upon illumination, untreated and acid-treated films exhibit opposite contraction and expansion behaviors relative to each other. When a narrow strip of a non-treated film is illuminated on one of its surfaces, contraction of the domains caused by the action of the dye occurs mainly in the illuminated surface layer, leading to rapid bending of the film along its long axis and toward the light source. The film returns to its initial flat state once the lamp is turned off. Nevertheless, when the protonated surface of an acid-treated **LCE-A20** strip is illuminated, the film contracts in the opposite direction, that is, toward the opposite side of the light source. In this case, since the first layers are already disordered, upon illumination, the action of the dye affects the orientation of the subsequent material layers. Therefore, this guides the movement toward the opposite side. Again, complete extension of the non-treated surface occurs by switching off the light source, recovering the original bent state.


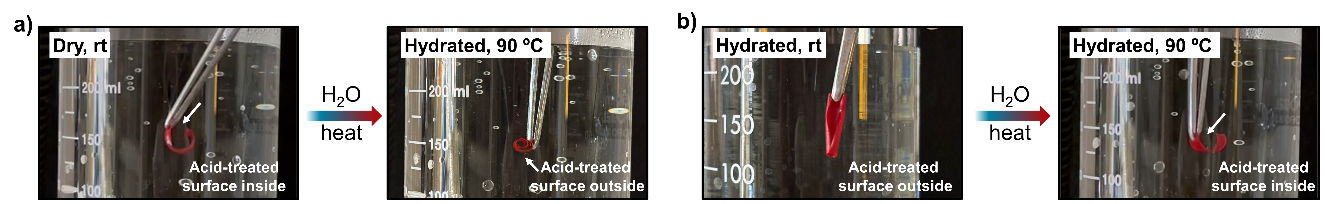


Figure S24. Second-stage actuation upon immersion in hot water of a) dry and b) hydrated acid-treated films. When the dry acid-treated film is submerged in hot water, it curls parallel to the alignment direction, while leaving the treated surface exposed outward. This behavior can possibly be explained by a combination of swelling of the treated surface and contraction of the untreated side, which remains on the inside of the curl. In contrast, when a film previously hydrated at room temperature (and therefore forming a tube) is immersed in hot water, the treated side expands so that tube structure disappears, and the film slightly bends toward the treated surface, i.e., leaving the acid-treated surface inwards. This change in behavior could occur because, after prior hydration, uneven swelling of the film is no longer the main factor driving the deformation. Instead, the thermal response becomes dominant: since the acid treated side of the film is already disordered, the effect of temperature is more pronounced on the untreated side, leading to its greater contraction.


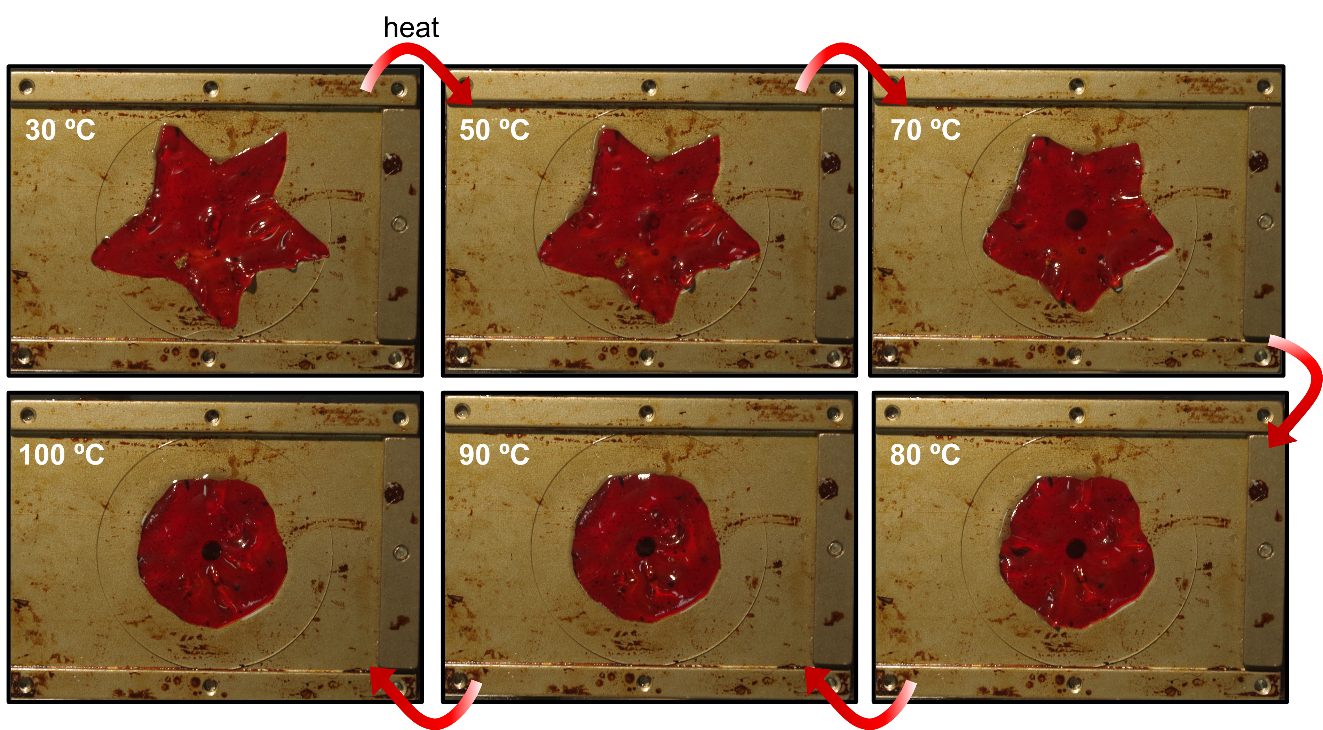


Figure S25. Gradual contraction upon heating of the star-shaped actuator (from 30 to 100 ºC).


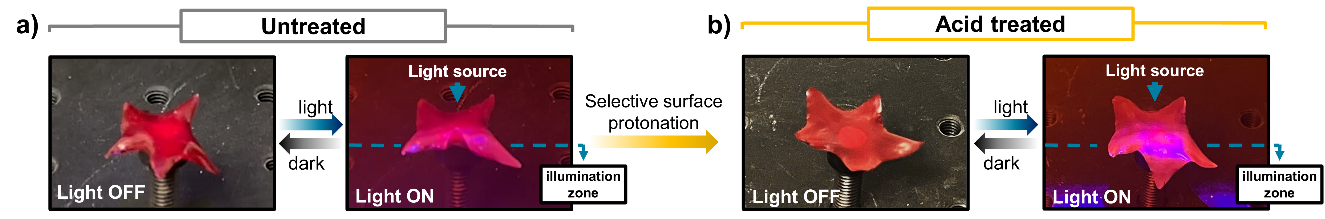


Figure S26. a) Orthogonal light actuation upon selective arm illumination of the untreated and b) acid treated star-shaped actuator. When one arm is selectively illuminated, both in the untreated star and the acid-treated star, the material exhibits distinct and clearly defined orthogonal bending directions, demonstrating the precise responsiveness of the actuator under controlled conditions.


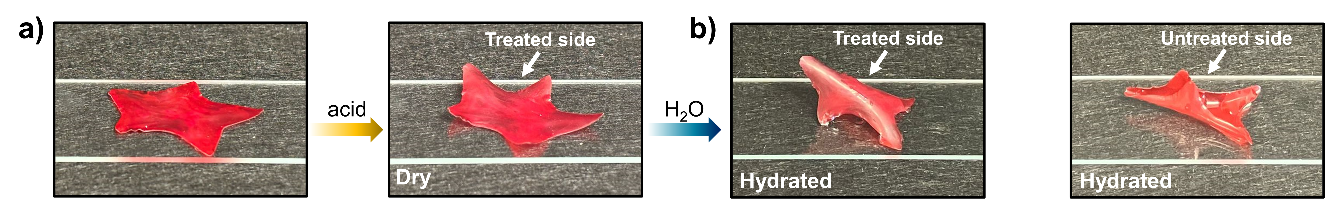


Figure S27. a) Star-shaped actuator before (left) and after (right) acid treatment of one of its surfaces. b) Acid treated star-shaped actuator after being immersed in water for 30 seconds, with the protonated surface facing upwards (left) and downwards (right). Upon water immersion, isotropic swelling of the protonated surface induces the bending of the material. The observed actuation, where two of the arms move toward each other until they make contact, can be attributed to an asymmetry in the stretching of the star's arms. In this case, this results in an average nematic director that traverses the upper arm, thereby guiding the bending around it.

**References**

[1] D. Martella, C. Parmeggiani, D. S. Wiersma, M. Piñol, L. Oriol, *J. Mater. Chem. C* **2015**, *3*, 9003–9010.

[2] P. J. Flory, *J. Am. Chem. Soc.* **1936**, *58*, 1877–1885.

[3] H. Liang, Y. Zhang, E. He, Y. Yang, Y. Liu, H. Xu, Z. Yang, Y. Wang, Y. Wei, Y. Ji, *Adv. Mater.* **2024**, *36*, 2400286.
